# Supplementary material for: Paternal family history of premature atherosclerotic disease and perinatal death: A population-based cohort study
Source: PLoS One. 2025 Jan 8;20(1):e0313821. doi: 10.1371/journal.pone.0313821 (PMC11709281; doi:10.1371/journal.pone.0313821)
Supplement: S1 File — (DOCX) [file pone.0313821.s001.docx]

## Supplementary Tables

Table S1.1 Number of fathers and births in different health surveys

| Health Survey | Number of fathers, n (%) | Number of births, n (%) |
| --- | --- | --- |
| CONOR | 32,891 (14.9) | 77,219 (15.0) |
| HU3 | 27,457 (12.4) | 54,686 (10.6) |
| HU40 | 161,013 (73.0) | 380,218 (74.0) |
| Total | 220,390 (100) | 512,123 (100) |

Table S1.2 Characteristics of mothers: overall and by fathers’ family history of coronary heart disease or stroke.^⊥^

| Maternal  Characteristics | | Fathers with information on family history of CHD | | |  | Fathers with information on family history of Stroke | | |
| --- | --- | --- | --- | --- | --- | --- | --- | --- |
|  |  | Total | No | Yes |  | Total | No | Yes |
| Number of Mothers, n (%) | | 236, 790 (100) | 208, 774 (88) | 29,840 (12) |  | 94,055 (100) | 85,504 (91) | 8,844(9) |
| No. births per mother, n (%) | |  |  |  |  |  |  |  |
|  | 1 | 45,900 (9.3)) | 40,397 (9.3) | 5,503 (10.8) |  | 16,569 (8.5) | 15,045 (8.5) | 1,524 (8.5) |
|  | 2-3 (reference) | 378,190 (76.6) | 331, 972(76.6) | 46,218 (76.6) |  | 150,359 (77.1) | 136,480 (77.0) | 13,879 (77.6) |
|  | ≥4 | 69,870 (14.1) | 61,215 (14.1) | 8,655 (14.3) |  | 28,151 (14.4) | 25,658 (14.5) | 2,493 (13.9) |
| ** Age at Childbirth in year,  mean (SD) | | 26.8 (5.8) | 26.9 (5.4) | 26.7 (5.3) |  | 27.5 (5.4) | 27.5 (5.4) | 27.3 (5.5) |
| **Age at Childbirth in year, n (%) | |  |  |  |  |  |  |  |
|  | ≤ 19 | 36,423 (7.11) | 31,494 (7.1) | 4,924 (7.9) |  | 12,600 (6.2) | 11,293 (6.1) | 1,307 (7.0) |
|  | 20-24 (reference) | 154,092 (30.1) | 135,169 (30.1) | 18,923 (30.2) |  | 52,743 (26.0) | 47,711 (25.9) | 5,032 (26.9) |
|  | 25 – 29 | 169,065 (33.0) | 148,276 (33.0) | 20,789 (33.1) |  | 68,051 (33.5) | 61,886 (33.5) | 6,165 (33.0) |
|  | 30 – 34 | 104,076 (20.32) | 91,330 (20.3) | 12,746 (20.3) |  | 47,266 (23.3) | 43,075 (23.3) | 4,191 (22.4) |
|  | ≥ 35 | 48, 455 (9.5) | 43,090 (9.6) | 5,365 (8.5) |  | 22,597 (11.1) | 20,585 (11.2) | 2,012 (10.8) |
| Parity, n (%) | |  |  |  |  |  |  |  |
|  | 0 | 203,059 (39.7) | 178,132(39.6) | 24,927 (39.7) |  | 82,440 (40.6) | 74,841 (40.6) | 7,599 (40.6) |
|  | 1 | 186,209 (36.7) | 163,422 (36.4) | 22,787 (36.3) |  | 73,454 (36.1) | 66,676 (36.1) | 6,778 (36.2) |
|  | ≥2 | 122,843 (24.0) | 107,805 (24.0) | 15,038 (24.0) |  | 47,363 (23.3) | 43,033 (23.3) | 4,330 (23.2) |
| **Marital Status, n (%) | |  |  |  |  |  |  |  |
|  | Married/Partner (reference) | 465,825 (91.0) | 410,007 (91.2) | 55,818 (89.0) |  | 181,510 (89.3) | 165,016 (89.4) | 16,494 (88.2) |
|  | Unmarried | 41,236 (8.1) | 34,918 (7.8) | 6,318 (10.1) |  | 19,732 (9.7) | 17,672 (9.6) | 2,060 (11.0) |
|  | Widowed/divorced/Separated | 5,049 (1.0) | 4,433 (1.0) | 616 (1.0) |  | 2,015 (1.0) | 1,862 (1.0) | 153 (0.8) |
| Diabetes Mellitus, n (%) | |  |  |  |  |  |  |  |
|  | No (reference) | 509,785 (99.6) | 447,315 (99.6) | 62,470 (99.6) |  | 201,781 (99.3) | 183,195 (99.3) | 18,586 (99.4) |
|  | Gestational diabetes | 1,120 (0.2) | 975 (0.2) | 145 (0.2) |  | 826 (0.4) | 761 (0.4) | 65 (0.4) |
|  | Pre-gestational diabetes | 1,206 (0.2) | 1,069 (0.2) | 137 (0.2) |  | 650 (0.3) | 594 (0.3) | 56 (0.3) |
| *^Preeclampsia, n (%) | |  |  |  |  |  |  |  |
|  | No | 498,474 (97.3) | 437,555 (97.4) | 60,919 (97.1) |  | 197,323 (97.1) | 179,169 (97.1) | 18,154 (97.0) |
|  | Yes | 13,637 (2.7) | 11,804 (2.6) | 1,833 (2.9) |  | 5,934 (2.9) | 5,381 (2.9) | 553 (3.0) |
| Chronic Hypertension, n (%) | |  |  |  |  |  |  |  |
|  | No | 510,922 (99.8) | 448,324 (99.8) | 62,598 (99.8) |  | 202,621 (99.7) | 183,978 (99.7) | 18,643 (99.7) |
|  | Yes | 1,189 (0.2) | 1,035 (0.2) | 154 (0.3) |  | 636 (0.3) | 572 (0.3) | 64 (0.3) |
| Placental abruptiop, n (%) | |  |  |  |  |  |  |  |
|  | No | 509,145 (99.4) | 446,760 (99.4) | 62,385 (99.4) |  | 202,072 (99.4) | 183,477 (99.4) | 18,595 (99.4) |
|  | Yes | 2,966 (0.6) | 2,599 (0.6) | 367 (0.6) |  | 1,185 (0.6) | 1,073 (0.6) | 112 (0.6) |
| Placenta Previa, n (%) | |  |  |  |  |  |  |  |
|  | No | 510,949 (99.8) | 448,330 (99.8) | 62,619 (99.8) |  | 202,826 (99.8) | 184,156 (99.8) | 18,670 (99.8) |
|  | Yes | 1,162 (0.2) | 1,029 (0.2) | 133 (0.2) |  | 431 (0.2) | 394 (0.2) | 37 (0.2) |

Difference between those with or without family history were tested by two sample t-test or Pearson chi2 tests.

** P value<0.05 for both CHD and Stroke

*^ P value<0.05 for CHD and P value> 0.05 for Stroke

@ including eclampsia and HELLP syndrome

^⊥^All this information is obtained from Medical Birth Registry of Norway

Table S1.3. Association between paternal family history of premature coronary heart disease and subsequent risk of perinatal death (excluding mothers with known risk factors of perinatal death) ^⊥^

| Family history of premature CHD | | Total | Deaths | ^Σ^Odds Ratio (95% CI) * |
| --- | --- | --- | --- | --- |
|  |  |  | N (%) |  |
| Stillbirth | | | | |
|  | No | 427,279 | 3,484 (0.8) | 1.00 (Reference) |
|  | Yes | 59,507 | 466 (0.8) | 1.01 (0.91; 1,12) |
| Neonatal Death | | | | |
|  | No | 423,795 | 1,980 (0.5) | 1.00 (Reference) |
|  | Yes | 59,041 | 256 (0.4) | 0.99 (0.86; 1.13) |
| Perinatal Death | | | | |
|  | No | 427,279 | 5,464 (1.3) | 1.00 (Reference) |
|  | Yes | 59,507 | 722 (1.2) | 1.00 (0.92; 1.09) |

^⊥^Excluding mothers with preeclampsia including HELLP syndrome and eclampsia, pregestational and gestational hypertension, pregestational and gestational diabetes

*Adjusted for year of birth, paternal [≤ 25, 26 – 30 (reference), 31 – 39, ≥ 40 yr] and maternal age [ ≤ 19, 20-24 (reference), 25 – 29, 30 – 34, ≥ 35] at childbirth, marital status married/have partner (reference), unmarried, widowed/divorced/ separated]

^Σ^Multilevel mixed effect logistic regression with random intercepts by father’s identification number. The birth is an observation unit.

Table S1.4. Association between paternal family history of premature stroke and subsequent risk of perinatal death (excluding mothers with known risk factors of perinatal death) ^⊥^

| Family history of premature CHD | | Total | Deaths | ^Σ^Odds Ratio (95% CI) * |
| --- | --- | --- | --- | --- |
|  |  |  | N (%) |  |
| Stillbirth | | | | |
|  | No | 174,534 | 1,173 (0.7) | 1.00 (Reference) |
|  | Yes | 17,696 | 116 (0.7) | 0.98 (0.80;1.20) |
| Neonatal Death | | | | |
|  | No | 173,361 | 575 (0.3) | 1.00 (Reference) |
|  | Yes | 17,580 | 66 (0.4) | 1.09 (0.84; 1.43) |
| Perinatal Death | | | | |
|  | No | 174,534 | 1,748(1.0) | 1.00 (Reference) |
|  | Yes | 17,696 | 182 (1.03) | 1.01 (0.86;1.19) |

^⊥^Excluding mothers with preeclampsia including HELLP syndrome and eclampsia, pregestational and gestational hypertension, pregestational and gestational diabetes

*Adjusted for year of birth, paternal [≤ 25, 26 – 30 (reference), 31 – 39, ≥ 40 yr] and maternal age [ ≤ 19, 20-24 (reference), 25 – 29, 30 – 34, ≥ 35] at childbirth, marital status married/have partner (reference), unmarried, widowed/divorced/ separated]

^Σ^Multilevel mixed effect logistic regression with random intercepts by father’s identification number. The birth is an observation unit.

Table S1.5: Association between paternal family history of premature coronary heart disease and subsequent risk of perinatal losses (restricted to the subpopulation with available data in father’s education)

| Family history of premature CHD | | Total | Deaths | ^Σ^Odds Ratio (95% CI) | |
| --- | --- | --- | --- | --- | --- |
|  |  |  | N (%) | Model 1* | Model 2** |
| Stillbirth | | | | | |
|  | No | 58,070 | 413 (0.7) | 1.00 (Ref) | 1.00 (Ref) |
|  | Yes | 9,578 | 60 (0.6) | 0.82 (0.62; 1.09) | 0.83 (0.62; 1.11) |
| Neonatal Death | | | | | |
|  | No | 57,657 | 158 (0.3) | 1.00 (Ref) | 1.00 (Ref) |
|  | Yes | 9,518 | 28 (0.3) | 0.96 (0.64; 1.45) | 0.97 (0.65; 1.47) |
| Perinatal Death | | | | | |
|  | No | 58,070 | 571 (1.0) | 1.00 (Ref) | 1.00 (Ref) |
|  | Yes | 9,578 | 88 (0.9) | 0.87 (0.68; 1.10) | 0.88 (0.69; 1.12) |

*Adjusted for birth year, paternal and maternal age at childbirth, and *paternal education*

**Adjusted for birth year, paternal and maternal age at childbirth, and *marital status*

^Σ^Multilevel mixed effect logistic regression with random intercepts by father’s identification number. The birth is an observation unit.

Table S1.6: Association between paternal family history of stroke and subsequent risk of perinatal losses (restricted to the subpopulation with available data on father’s education)

| Family history of stroke | | Total | Deaths | ^Σ^Odds Ratio (95% CI) | |
| --- | --- | --- | --- | --- | --- |
|  |  |  | N (%) | Model 1* | Model 2** |
| Stillbirth | | | | | |
|  | No | 61,888 | 432 (0.7) | 1.00 (Reference) | 1.00 (Reference) |
|  | Yes | 5,760 | 41 (0.7) | 0.99 (0.71; 1.39) | 0.99 (0.71; 1.40) |
| Neonatal Death | | | | | |
|  | No | 61,456 | 169 (0.3) | 1.00 (Reference) | 1.00 (Reference) |
|  | Yes | 5,719 | 17 (0.3) | 1.01 (0.61; 1.69) | 1.01 (0.61; 1.69) |
| Perinatal Death | | | | | |
|  | No | 61,888 | 601 (1.0) | 1.00 (Reference) | 1.00 (Reference) |
|  | Yes | 5,760 | 58 (1.0) | 1.00 (0.75; 1.33) | 1.00 (0.75; 1.33) |

*Adjusted for birth year, paternal and maternal age at childbirth, and *paternal education*

**Adjusted for birth year, paternal and maternal age at childbirth, and *marital status*

^Σ^Multilevel mixed effect logistic regression with random intercepts by father’s identification number. The birth is an observation unit.
