# Supplementary material for: Paternal family history of premature atherosclerotic disease and perinatal death: A population-based cohort study
Source: PLoS One. 2025 Jan 8;20(1):e0313821. doi: 10.1371/journal.pone.0313821 (PMC11709281; doi:10.1371/journal.pone.0313821)
Supplement: S2 File — (DOCX) [file pone.0313821.s002.docx]

Regression Output

-----------------------------------------------------------------------------------------------------

name: <unnamed>

log: S:\Project\ShweSin\family history\do file\Family History_Regressions_ Logfile.log

log type: text

opened on: 11 Mar 2024, 10:37:22

Regression S2.1. Paternal family history of premature CHD and Stillbirth

Model 1

. melogit fetalloss_total fam_chd60_final_f_v2 i.FAAR_K4 || FAR_LOPENR: , or

Mixed-effects logistic regression Number of obs = 512,111

Group variable: FAR_LOPENR Number of groups = 220,386

Obs per group:

min = 1

avg = 2.3

max = 15

Integration method: mvaghermite Integration pts. = 7

Wald chi2(4) = 88.79

Log likelihood = -24755.016 Prob > chi2 = 0.0000

--------------------------------------------------------------------------------------

fetalloss_total | Odds ratio Std. err. z P>|z| [95% conf. interval]

---------------------+----------------------------------------------------------------

fam_chd60_final_f_v2 | .9773886 .0485933 -0.46 0.646 .8866408 1.077425

|

FAAR_K4 |

1978_1988 | .722981 .0260173 -9.01 0.000 .6737448 .7758153

1989_1999 | .891002 .0393538 -2.61 0.009 .8171143 .971571

2000_max | .7083492 .0606468 -4.03 0.000 .5989217 .8377699

|

_cons | .0046792 .0002529 -99.25 0.000 .0042089 .0052022

---------------------+----------------------------------------------------------------

FAR_LOPENR |

var(_cons)| 1.554925 .1003746 1.370131 1.764643

--------------------------------------------------------------------------------------

Note: Estimates are transformed only in the first equation to odds ratios.

Note: _cons estimates baseline odds (conditional on zero random effects).

LR test vs. logistic model: chibar2(01) = 271.73 Prob >= chibar2 = 0.0000

. estat icc

Residual intraclass correlation

------------------------------------------------------------------------------

Level | ICC Std. err. [95% conf. interval]

-----------------------------+------------------------------------------------

FAR_LOPENR | .3209477 .0140686 .2940196 .3491224

------------------------------------------------------------------------------

.

. estimates store m1

.

Model 2

. melogit fetalloss_total fam_chd60_final_f_v2 i.FAAR_K4 i.fAge_KAT_K4 i.MORS_ALDER_K5 i.mCivil ||

> FAR_LOPENR: , or

Mixed-effects logistic regression Number of obs = 512,110

Group variable: FAR_LOPENR Number of groups = 220,386

Obs per group:

min = 1

avg = 2.3

max = 15

Integration method: mvaghermite Integration pts. = 7

Wald chi2(13) = 382.33

Log likelihood = -24595.11 Prob > chi2 = 0.0000

--------------------------------------------------------------------------------------

fetalloss_total | Odds ratio Std. err. z P>|z| [95% conf. interval]

---------------------+----------------------------------------------------------------

fam_chd60_final_f_v2 | 1.01555 .0505581 0.31 0.757 .9211391 1.119638

|

FAAR_K4 |

1978_1988 | .6572005 .0268264 -10.28 0.000 .60667 .7119398

1989_1999 | .6200554 .0346203 -8.56 0.000 .5557819 .6917619

2000_max | .4440586 .0425547 -8.47 0.000 .3680176 .5358114

|

fAge_KAT_K4 |

min-25 | .9205694 .0471479 -1.62 0.106 .8326479 1.017775

31-39 | 1.071364 .0526027 1.40 0.160 .9730702 1.179587

40-max | 1.202196 .0946876 2.34 0.019 1.030227 1.402871

|

MORS_ALDER_K5 |

min_19 | 1.049935 .0745496 0.69 0.493 .9135315 1.206705

25-29 | .9731618 .0467987 -0.57 0.572 .8856281 1.069347

30-34 | 1.140072 .0706173 2.12 0.034 1.009737 1.287232

35-max | 1.772115 .1303395 7.78 0.000 1.534214 2.046907

|

mCivil |

unmarried | .4177632 .0363812 -10.02 0.000 .3522109 .4955159

wdo_divo_sep | .7800979 .1344147 -1.44 0.150 .5565227 1.093491

|

_cons | .0050916 .0003372 -79.72 0.000 .0044717 .0057974

---------------------+----------------------------------------------------------------

FAR_LOPENR |

var(_cons)| 1.510789 .0994659 1.327893 1.718875

--------------------------------------------------------------------------------------

Note: Estimates are transformed only in the first equation to odds ratios.

Note: _cons estimates baseline odds (conditional on zero random effects).

LR test vs. logistic model: chibar2(01) = 259.65 Prob >= chibar2 = 0.0000

.

end of do-file

. do "C:\Users\SHWIN8~1\AppData\Local\Temp\STD172c_000000.tmp"

. estat icc

Residual intraclass correlation

------------------------------------------------------------------------------

Level | ICC Std. err. [95% conf. interval]

-----------------------------+------------------------------------------------

FAR_LOPENR | .3147046 .0141988 .2875621 .3431749

------------------------------------------------------------------------------

.

end of do-file

.

. estimates store m2

.

end of do-file

. do "C:\Users\SHWIN8~1\AppData\Local\Temp\STD172c_000000.tmp"

. estimates stats m1 m2

Akaike's information criterion and Bayesian information criterion

-----------------------------------------------------------------------------

Model | N ll(null) ll(model) df AIC BIC

-------------+---------------------------------------------------------------

m1 | 512,111 . -24755.02 6 49522.03 49588.91

m2 | 512,110 . -24595.11 15 49220.22 49387.42

-----------------------------------------------------------------------------

Note: BIC uses N = number of observations. See [R] IC note.

.

end of do-file

. do "C:\Users\SHWIN8~1\AppData\Local\Temp\STD172c_000000.tmp"

. lrtest m1 m2, force

Likelihood-ratio test

Assumption: m1 nested within m2

LR chi2(9) = 319.81

Prob > chi2 = 0.0000

.

. estimates clear

Regression S2.2. Paternal family history of premature CHD and neonatal Death

Model 1

. melogit neo fam_chd60_final_f_v2 i.FAAR_K4 || FAR_LOPENR: , or

Mixed-effects logistic regression Number of obs = 507,790

Group variable: FAR_LOPENR Number of groups = 220,178

Obs per group:

min = 1

avg = 2.3

max = 15

Integration method: mvaghermite Integration pts. = 7

Wald chi2(4) = 301.59

Log likelihood = -14933.947 Prob > chi2 = 0.0000

--------------------------------------------------------------------------------------

neo | Odds ratio Std. err. z P>|z| [95% conf. interval]

---------------------+----------------------------------------------------------------

fam_chd60_final_f_v2 | .9724045 .0651905 -0.42 0.676 .852672 1.10895

|

FAAR_K4 |

1978_1988 | .5447336 .025128 -13.17 0.000 .4976444 .5962787

1989_1999 | .4350943 .0293171 -12.35 0.000 .3812665 .4965215

2000_max | .2251407 .0385069 -8.72 0.000 .1610159 .3148033

|

_cons | .0030529 .000251 -70.45 0.000 .0025985 .0035866

---------------------+----------------------------------------------------------------

FAR_LOPENR |

var(_cons)| 1.706082 .1600089 1.419607 2.050368

--------------------------------------------------------------------------------------

Note: Estimates are transformed only in the first equation to odds ratios.

Note: _cons estimates baseline odds (conditional on zero random effects).

LR test vs. logistic model: chibar2(01) = 115.13 Prob >= chibar2 = 0.0000

. estat icc

Residual intraclass correlation

------------------------------------------------------------------------------

Level | ICC Std. err. [95% conf. interval]

-----------------------------+------------------------------------------------

FAR_LOPENR | .341493 .0210905 .3014363 .3839471

------------------------------------------------------------------------------

. estimates store m1

Model 2

. melogit neo fam_chd60_final_f_v2 i.FAAR_K4 i.fAge_KAT_K4 i.MORS_ALDER_K5 i.mCivil || FAR_LOPENR:

> , or

Mixed-effects logistic regression Number of obs = 507,789

Group variable: FAR_LOPENR Number of groups = 220,178

Obs per group:

min = 1

avg = 2.3

max = 15

Integration method: mvaghermite Integration pts. = 7

Wald chi2(13) = 372.17

Log likelihood = -14900.94 Prob > chi2 = 0.0000

--------------------------------------------------------------------------------------

neo | Odds ratio Std. err. z P>|z| [95% conf. interval]

---------------------+----------------------------------------------------------------

fam_chd60_final_f_v2 | .9762786 .065615 -0.36 0.721 .8557859 1.113736

|

FAAR_K4 |

1978_1988 | .588623 .031032 -10.05 0.000 .5308381 .652698

1989_1999 | .420138 .0347562 -10.48 0.000 .357253 .4940924

2000_max | .2034888 .0371485 -8.72 0.000 .142281 .2910275

|

fAge_KAT_K4 |

min-25 | 1.04795 .0663409 0.74 0.459 .9256674 1.186387

31-39 | 1.071345 .0726356 1.02 0.309 .9380348 1.2236

40-max | 1.279753 .1552177 2.03 0.042 1.008989 1.623177

|

MORS_ALDER_K5 |

min_19 | 1.488925 .1101847 5.38 0.000 1.287898 1.72133

25-29 | .8985301 .055758 -1.72 0.085 .7956309 1.014737

30-34 | .9554663 .0810829 -0.54 0.591 .8090597 1.128367

35-max | 1.110534 .1234031 0.94 0.345 .8931944 1.380759

|

mCivil |

unmarried | .6524964 .0571436 -4.88 0.000 .5495821 .7746825

wdo_divo_sep | .8841126 .2080278 -0.52 0.601 .5574726 1.402141

|

_cons | .0029012 .0002821 -60.08 0.000 .0023977 .0035105

---------------------+----------------------------------------------------------------

FAR_LOPENR |

var(_cons)| 1.711897 .1607083 1.424194 2.057719

--------------------------------------------------------------------------------------

Note: Estimates are transformed only in the first equation to odds ratios.

Note: _cons estimates baseline odds (conditional on zero random effects).

LR test vs. logistic model: chibar2(01) = 115.29 Prob >= chibar2 = 0.0000

.

. estat icc

Residual intraclass correlation

------------------------------------------------------------------------------

Level | ICC Std. err. [95% conf. interval]

-----------------------------+------------------------------------------------

FAR_LOPENR | .3422585 .0211334 .302116 .384794

------------------------------------------------------------------------------

.

. estimates store m2

.

.

. estimates stats m1 m2

Akaike's information criterion and Bayesian information criterion

-----------------------------------------------------------------------------

Model | N ll(null) ll(model) df AIC BIC

-------------+---------------------------------------------------------------

m1 | 507,790 . -14933.95 6 29879.89 29946.72

m2 | 507,789 . -14900.94 15 29831.88 29998.95

-----------------------------------------------------------------------------

Note: BIC uses N = number of observations. See [R] IC note.

. lrtest m1 m2, force

Likelihood-ratio test

Assumption: m1 nested within m2

LR chi2(9) = 66.01

Prob > chi2 = 0.0000

.

. estimates clear

Regression S2.3. Paternal family history of premature CHD and perinatal Death

Model 1

. melogit totalloss_12aug fam_chd60_final_f_v2 i.FAAR_K4 || FAR_LOPENR: , or

Mixed-effects logistic regression Number of obs = 512,111

Group variable: FAR_LOPENR Number of groups = 220,386

Obs per group:

min = 1

avg = 2.3

max = 15

Integration method: mvaghermite Integration pts. = 7

Wald chi2(4) = 291.38

Log likelihood = -35349.334 Prob > chi2 = 0.0000

--------------------------------------------------------------------------------------

totalloss_12aug | Odds ratio Std. err. z P>|z| [95% conf. interval]

---------------------+----------------------------------------------------------------

fam_chd60_final_f_v2 | .9754777 .0397303 -0.61 0.542 .9006347 1.05654

|

FAAR_K4 |

1978_1988 | .6446695 .0185397 -15.27 0.000 .6093375 .6820501

1989_1999 | .6972617 .0258748 -9.72 0.000 .6483484 .7498651

2000_max | .5044751 .0386685 -8.93 0.000 .4341045 .5862532

|

_cons | .008661 .0003393 -121.21 0.000 .0080208 .0093524

---------------------+----------------------------------------------------------------

FAR_LOPENR |

var(_cons)| 1.413457 .0729037 1.277553 1.563817

--------------------------------------------------------------------------------------

Note: Estimates are transformed only in the first equation to odds ratios.

Note: _cons estimates baseline odds (conditional on zero random effects).

LR test vs. logistic model: chibar2(01) = 458.05 Prob >= chibar2 = 0.0000

. estat icc

Residual intraclass correlation

------------------------------------------------------------------------------

Level | ICC Std. err. [95% conf. interval]

-----------------------------+------------------------------------------------

FAR_LOPENR | .3005229 .0108422 .27971 .3221918

------------------------------------------------------------------------------

. estimates store m1

.

Model 2

. melogit totalloss_12aug fam_chd60_final_f_v2 i.FAAR_K4 i.fAge_KAT_K4 i.MORS_ALDER_K5 i.mCivil ||

> FAR_LOPENR: , or

Mixed-effects logistic regression Number of obs = 512,110

Group variable: FAR_LOPENR Number of groups = 220,386

Obs per group:

min = 1

avg = 2.3

max = 15

Integration method: mvaghermite Integration pts. = 7

Wald chi2(13) = 574.98

Log likelihood = -35196.322 Prob > chi2 = 0.0000

--------------------------------------------------------------------------------------

totalloss_12aug | Odds ratio Std. err. z P>|z| [95% conf. interval]

---------------------+----------------------------------------------------------------

fam_chd60_final_f_v2 | 1.001662 .040863 0.04 0.968 .9246901 1.085041

|

FAAR_K4 |

1978_1988 | .6270972 .0205426 -14.25 0.000 .5880999 .6686806

1989_1999 | .5454779 .0254888 -12.97 0.000 .4977402 .5977942

2000_max | .3593084 .0304818 -12.07 0.000 .3042678 .4243055

|

fAge_KAT_K4 |

min-25 | .9732939 .0392384 -0.67 0.502 .899348 1.05332

31-39 | 1.070522 .0430827 1.69 0.090 .9893262 1.158382

40-max | 1.222088 .0816274 3.00 0.003 1.072131 1.393019

|

MORS_ALDER_K5 |

min_19 | 1.246757 .0645493 4.26 0.000 1.12645 1.379913

25-29 | .9446394 .0363472 -1.48 0.139 .8760201 1.018634

30-34 | 1.074266 .0543615 1.42 0.157 .9728325 1.186275

35-max | 1.547552 .0955582 7.07 0.000 1.371151 1.746648

|

mCivil |

unmarried | .50808 .0315425 -10.91 0.000 .4498709 .5738207

wdo_divo_sep | .8128531 .1142529 -1.47 0.140 .6171191 1.070669

|

_cons | .0089016 .0004465 -94.14 0.000 .0080682 .0098211

---------------------+----------------------------------------------------------------

FAR_LOPENR |

var(_cons)| 1.388651 .0726112 1.253385 1.538514

--------------------------------------------------------------------------------------

Note: Estimates are transformed only in the first equation to odds ratios.

Note: _cons estimates baseline odds (conditional on zero random effects).

LR test vs. logistic model: chibar2(01) = 444.23 Prob >= chibar2 = 0.0000

. estat icc

Residual intraclass correlation

------------------------------------------------------------------------------

Level | ICC Std. err. [95% conf. interval]

-----------------------------+------------------------------------------------

FAR_LOPENR | .2968142 .0109135 .2758783 .3186397

------------------------------------------------------------------------------

. estimates store m2

. estimates stats m1 m2

Akaike's information criterion and Bayesian information criterion

-----------------------------------------------------------------------------

Model | N ll(null) ll(model) df AIC BIC

-------------+---------------------------------------------------------------

m1 | 512,111 . -35349.33 6 70710.67 70777.55

m2 | 512,110 . -35196.32 15 70422.64 70589.84

-----------------------------------------------------------------------------

Note: BIC uses N = number of observations. See [R] IC note.

. lrtest m1 m2, force

Likelihood-ratio test

Assumption: m1 nested within m2

LR chi2(9) = 306.02

Prob > chi2 = 0.0000

. estimates clear

Regression S2.4. Paternal family history of premature stroke and stillbirth

Model 1

. melogit fetalloss_total fam_stroke70_final_f_v2 i.FAAR_K4 || FAR_LOPENR: , or

Mixed-effects logistic regression Number of obs = 203,257

Group variable: FAR_LOPENR Number of groups = 85,428

Obs per group:

min = 1

avg = 2.4

max = 13

Integration method: mvaghermite Integration pts. = 7

Wald chi2(4) = 32.18

Log likelihood = -8141.7735 Prob > chi2 = 0.0000

-----------------------------------------------------------------------------------------

fetalloss_total | Odds ratio Std. err. z P>|z| [95% conf. interval]

------------------------+----------------------------------------------------------------

fam_stroke70_final_f_v2 | 1.001163 .0995738 0.01 0.991 .8238458 1.216645

|

FAAR_K4 |

1978_1988 | .8241512 .0895874 -1.78 0.075 .6660073 1.019846

1989_1999 | 1.170702 .1280444 1.44 0.150 .9448153 1.450593

2000_max | .9805981 .131036 -0.15 0.883 .754651 1.274195

|

_cons | .0028007 .0003714 -44.32 0.000 .0021597 .003632

------------------------+----------------------------------------------------------------

FAR_LOPENR |

var(_cons)| 1.853571 .1828825 1.527656 2.249019

-----------------------------------------------------------------------------------------

Note: Estimates are transformed only in the first equation to odds ratios.

Note: _cons estimates baseline odds (conditional on zero random effects).

LR test vs. logistic model: chibar2(01) = 124.50 Prob >= chibar2 = 0.0000

. estat icc

Residual intraclass correlation

------------------------------------------------------------------------------

Level | ICC Std. err. [95% conf. interval]

-----------------------------+------------------------------------------------

FAR_LOPENR | .3603759 .0227428 .317104 .4060416

------------------------------------------------------------------------------

. estimates store m1

.

Model 2

. melogit fetalloss_total fam_stroke70_final_f_v2 i.FAAR_K4 i.fAge_KAT_K4 i.MORS_ALDER_K5 i.mCivil|

> | FAR_LOPENR: , or

Mixed-effects logistic regression Number of obs = 203,257

Group variable: FAR_LOPENR Number of groups = 85,428

Obs per group:

min = 1

avg = 2.4

max = 13

Integration method: mvaghermite Integration pts. = 7

Wald chi2(13) = 132.01

Log likelihood = -8090.3743 Prob > chi2 = 0.0000

-----------------------------------------------------------------------------------------

fetalloss_total | Odds ratio Std. err. z P>|z| [95% conf. interval]

------------------------+----------------------------------------------------------------

fam_stroke70_final_f_v2 | .9972652 .0991796 -0.03 0.978 .8206487 1.211892

|

FAAR_K4 |

1978_1988 | .7708328 .0982672 -2.04 0.041 .6004093 .9896302

1989_1999 | .8251247 .1215134 -1.31 0.192 .6182526 1.101218

2000_max | .6345534 .1104609 -2.61 0.009 .4511218 .8925704

|

fAge_KAT_K4 |

min-25 | .9872727 .1020595 -0.12 0.901 .8062023 1.209011

31-39 | 1.098902 .101509 1.02 0.307 .916919 1.317004

40-max | .9667898 .13601 -0.24 0.810 .7338093 1.27374

|

MORS_ALDER_K5 |

min_19 | 1.01034 .1490547 0.07 0.944 .7566424 1.349102

25-29 | .819219 .0752508 -2.17 0.030 .6842446 .9808185

30-34 | 1.095114 .1194764 0.83 0.405 .884288 1.356204

35-max | 1.750976 .219522 4.47 0.000 1.369506 2.238702

|

mCivil |

unmarried | .4757565 .0647731 -5.46 0.000 .3643307 .6212605

wdo_divo_sep | 1.175453 .2990731 0.64 0.525 .7138918 1.935434

|

_cons | .0034251 .0005801 -33.51 0.000 .0024576 .0047737

------------------------+----------------------------------------------------------------

FAR_LOPENR |

var(_cons)| 1.805858 .1816156 1.482785 2.199324

-----------------------------------------------------------------------------------------

Note: Estimates are transformed only in the first equation to odds ratios.

Note: _cons estimates baseline odds (conditional on zero random effects).

LR test vs. logistic model: chibar2(01) = 119.26 Prob >= chibar2 = 0.0000

. estat icc

Residual intraclass correlation

------------------------------------------------------------------------------

Level | ICC Std. err. [95% conf. interval]

-----------------------------+------------------------------------------------

FAR_LOPENR | .3543868 .0230101 .3106836 .4006644

------------------------------------------------------------------------------

. estimates store m2

. estimates stats m1 m2

Akaike's information criterion and Bayesian information criterion

-----------------------------------------------------------------------------

Model | N ll(null) ll(model) df AIC BIC

-------------+---------------------------------------------------------------

m1 | 203,257 . -8141.773 6 16295.55 16356.88

m2 | 203,257 . -8090.374 15 16210.75 16364.08

-----------------------------------------------------------------------------

Note: BIC uses N = number of observations. See [R] IC note.

. lrtest m1 m2, force

Likelihood-ratio test

Assumption: m1 nested within m2

LR chi2(9) = 102.80

Prob > chi2 = 0.0000

. estimates clear .

Regression S2.5. Paternal family history of premature stroke and neonatal death

Model 1

. melogit neo fam_stroke70_final_f_v2 i.FAAR_K4 || FAR_LOPENR: , or

Mixed-effects logistic regression Number of obs = 201,886

Group variable: FAR_LOPENR Number of groups = 85,383

Obs per group:

min = 1

avg = 2.4

max = 12

Integration method: mvaghermite Integration pts. = 7

Wald chi2(4) = 73.33

Log likelihood = -4508.1678 Prob > chi2 = 0.0000

-----------------------------------------------------------------------------------------

neo | Odds ratio Std. err. z P>|z| [95% conf. interval]

------------------------+----------------------------------------------------------------

fam_stroke70_final_f_v2 | 1.09288 .1431289 0.68 0.498 .8454639 1.4127

|

FAAR_K4 |

1978_1988 | .5429174 .0626223 -5.30 0.000 .4330646 .6806359

1989_1999 | .4011584 .0508243 -7.21 0.000 .3129493 .5142306

2000_max | .2397879 .0488791 -7.01 0.000 .1608109 .3575517

|

_cons | .0026963 .0004852 -32.87 0.000 .0018948 .0038367

------------------------+----------------------------------------------------------------

FAR_LOPENR |

var(_cons)| 1.844461 .313653 1.321666 2.574053

-----------------------------------------------------------------------------------------

Note: Estimates are transformed only in the first equation to odds ratios.

Note: _cons estimates baseline odds (conditional on zero random effects).

LR test vs. logistic model: chibar2(01) = 33.83 Prob >= chibar2 = 0.0000

. estat icc

Residual intraclass correlation

------------------------------------------------------------------------------

Level | ICC Std. err. [95% conf. interval]

-----------------------------+------------------------------------------------

FAR_LOPENR | .359241 .0391436 .2866001 .4389645

------------------------------------------------------------------------------

. estimates store m1

.

end of do-file

Model 2

. melogit neo fam_stroke70_final_f_v2 i.FAAR_K4 i.fAge_KAT_K4 i.MORS_ALDER_K5 i.mCivil || FAR_LOPE

> NR: , or

Iteration 4: Log likelihood = -4500.7608

Mixed-effects logistic regression Number of obs = 201,886

Group variable: FAR_LOPENR Number of groups = 85,383

Obs per group:

min = 1

avg = 2.4

max = 12

Integration method: mvaghermite Integration pts. = 7

Wald chi2(13) = 89.98

Log likelihood = -4500.7608 Prob > chi2 = 0.0000

-----------------------------------------------------------------------------------------

neo | Odds ratio Std. err. z P>|z| [95% conf. interval]

------------------------+----------------------------------------------------------------

fam_stroke70_final_f_v2 | 1.090317 .1429203 0.66 0.509 .843288 1.409709

|

FAAR_K4 |

1978_1988 | .6395896 .0889622 -3.21 0.001 .4869737 .8400347

1989_1999 | .4786626 .0850906 -4.14 0.000 .337842 .6781805

2000_max | .276673 .0706037 -5.04 0.000 .1677839 .4562297

|

fAge_KAT_K4 |

min-25 | 1.076674 .1368819 0.58 0.561 .8392036 1.381341

31-39 | .9771326 .1262603 -0.18 0.858 .7585172 1.258756

40-max | 1.093586 .251057 0.39 0.697 .6973341 1.715004

|

MORS_ALDER_K5 |

min_19 | 1.41938 .2163076 2.30 0.022 1.05288 1.913455

25-29 | .9205488 .1084108 -0.70 0.482 .7308072 1.159553

30-34 | .9372197 .1445877 -0.42 0.674 .6926663 1.268115

35-max | .989351 .2012034 -0.05 0.958 .6641136 1.473868

|

mCivil |

unmarried | .6648026 .0955611 -2.84 0.005 .5015774 .8811452

wdo_divo_sep | .6274452 .3190106 -0.92 0.359 .2316335 1.699614

|

_cons | .0024074 .0005423 -26.77 0.000 .0015482 .0037435

------------------------+----------------------------------------------------------------

FAR_LOPENR |

var(_cons)| 1.847376 .3142947 1.323549 2.57852

-----------------------------------------------------------------------------------------

Note: Estimates are transformed only in the first equation to odds ratios.

Note: _cons estimates baseline odds (conditional on zero random effects).

LR test vs. logistic model: chibar2(01) = 33.82 Prob >= chibar2 = 0.0000

. estat icc

Residual intraclass correlation

------------------------------------------------------------------------------

Level | ICC Std. err. [95% conf. interval]

-----------------------------+------------------------------------------------

FAR_LOPENR | .3596045 .0391792 .2868913 .4393915

------------------------------------------------------------------------------

. estimates store m2

. estimates stats m1 m2

Akaike's information criterion and Bayesian information criterion

-----------------------------------------------------------------------------

Model | N ll(null) ll(model) df AIC BIC

-------------+---------------------------------------------------------------

m1 | 201,886 . -4508.168 6 9028.336 9089.628

m2 | 201,886 . -4500.761 15 9031.522 9184.754

-----------------------------------------------------------------------------

Note: BIC uses N = number of observations. See [R] IC note.

. lrtest m1 m2, force

Likelihood-ratio test

Assumption: m1 nested within m2

LR chi2(9) = 14.81

Prob > chi2 = 0.0962

. estimates clear

.

end of do-file

Regression S2.6. Paternal family history of premature stroke and perinatal death

Model 1

. melogit totalloss_12aug fam_stroke70_final_f_v2 i.FAAR_K4 || FAR_LOPENR: , or

Iteration 3: Log likelihood = -11362.092

Mixed-effects logistic regression Number of obs = 203,257

Group variable: FAR_LOPENR Number of groups = 85,428

Obs per group:

min = 1

avg = 2.4

max = 13

Integration method: mvaghermite Integration pts. = 7

Wald chi2(4) = 31.75

Log likelihood = -11362.092 Prob > chi2 = 0.0000

-----------------------------------------------------------------------------------------

totalloss_12aug | Odds ratio Std. err. z P>|z| [95% conf. interval]

------------------------+----------------------------------------------------------------

fam_stroke70_final_f_v2 | 1.023579 .0832055 0.29 0.774 .8728273 1.200368

|

FAAR_K4 |

1978_1988 | .6791628 .0546192 -4.81 0.000 .5801217 .7951128

1989_1999 | .7841221 .0647909 -2.94 0.003 .6668837 .9219712

2000_max | .6094876 .0660091 -4.57 0.000 .4929212 .7536196

|

_cons | .0059957 .0005787 -53.01 0.000 .0049623 .0072444

------------------------+----------------------------------------------------------------

FAR_LOPENR |

var(_cons)| 1.71152 .1375064 1.462161 2.003406

-----------------------------------------------------------------------------------------

Note: Estimates are transformed only in the first equation to odds ratios.

Note: _cons estimates baseline odds (conditional on zero random effects).

LR test vs. logistic model: chibar2(01) = 198.36 Prob >= chibar2 = 0.0000

. estat icc

Residual intraclass correlation

------------------------------------------------------------------------------

Level | ICC Std. err. [95% conf. interval]

-----------------------------+------------------------------------------------

FAR_LOPENR | .342209 .0180851 .3076919 .3784814

------------------------------------------------------------------------------

. estimates store m1

.

end of do-file

Model 2

. melogit totalloss_12aug fam_stroke70_final_f_v2 i.FAAR_K4 i.fAge_KAT_K4 i.MORS_ALDER_K5 i.mCivil

> || FAR_LOPENR: , or

Mixed-effects logistic regression Number of obs = 203,257

Group variable: FAR_LOPENR Number of groups = 85,428

Obs per group:

min = 1

avg = 2.4

max = 13

Integration method: mvaghermite Integration pts. = 7

Wald chi2(13) = 120.77

Log likelihood = -11316.332 Prob > chi2 = 0.0000

-----------------------------------------------------------------------------------------

totalloss_12aug | Odds ratio Std. err. z P>|z| [95% conf. interval]

------------------------+----------------------------------------------------------------

fam_stroke70_final_f_v2 | 1.02044 .0829696 0.25 0.803 .8701182 1.196732

|

FAAR_K4 |

1978_1988 | .7027688 .0670493 -3.70 0.000 .5829101 .847273

1989_1999 | .6718934 .0765157 -3.49 0.000 .5374832 .839916

2000_max | .4865827 .0687733 -5.10 0.000 .3688489 .6418964

|

fAge_KAT_K4 |

min-25 | 1.026791 .083362 0.33 0.745 .8757407 1.203894

31-39 | 1.056177 .0801554 0.72 0.471 .910201 1.225564

40-max | .9800358 .1183523 -0.17 0.867 .7734782 1.241755

|

MORS_ALDER_K5 |

min_19 | 1.198463 .1283013 1.69 0.091 .9716269 1.478257

25-29 | .8550537 .06278 -2.13 0.033 .7404509 .9873941

30-34 | 1.050535 .0945622 0.55 0.584 .8806248 1.253228

35-max | 1.54158 .1643685 4.06 0.000 1.250858 1.899871

|

mCivil |

unmarried | .5478323 .0546142 -6.04 0.000 .4505988 .6660475

wdo_divo_sep | .9919885 .2278844 -0.04 0.972 .6323601 1.55614

|

_cons | .0063792 .0008169 -39.47 0.000 .0049633 .0081991

------------------------+----------------------------------------------------------------

FAR_LOPENR |

var(_cons)| 1.686491 .1370554 1.438168 1.97769

-----------------------------------------------------------------------------------------

Note: Estimates are transformed only in the first equation to odds ratios.

Note: _cons estimates baseline odds (conditional on zero random effects).

LR test vs. logistic model: chibar2(01) = 193.29 Prob >= chibar2 = 0.0000

. estat icc

Residual intraclass correlation

------------------------------------------------------------------------------

Level | ICC Std. err. [95% conf. interval]

-----------------------------+------------------------------------------------

FAR_LOPENR | .3389005 .0182075 .3041787 .3754472

------------------------------------------------------------------------------

. estimates store m2

. estimates stats m1 m2

Akaike's information criterion and Bayesian information criterion

-----------------------------------------------------------------------------

Model | N ll(null) ll(model) df AIC BIC

-------------+---------------------------------------------------------------

m1 | 203,257 . -11362.09 6 22736.18 22797.52

m2 | 203,257 . -11316.33 15 22662.66 22816

-----------------------------------------------------------------------------

Note: BIC uses N = number of observations. See [R] IC note.

. lrtest m1 m2, force

Likelihood-ratio test

Assumption: m1 nested within m2

LR chi2(9) = 91.52

Prob > chi2 = 0.0000

. estimates clear

.

end of do-file

. log close

name: <unnamed>

log: S:\Project\ShweSin\family history\do file\Family History_Regressions_ Logfile.log

log type: text

closed on: 11 Mar 2024, 11:24:58

Additional analysis (excluding mother with risk factors of perinatal death)

**Family history of CHD**

Regression S2.7. Paternal family history of premature CHD and stillbirth (excluding mother with risk factors of perinatal death)

. melogit fetalloss_total fam_chd60_final_f_v2 i.FAAR_K4 i.fAge_KAT_K4 i.MORS_ALDER_K5 i.mCivil if preeclampsia_ext==0 & HYPERTENSJON_KRONISK ==0 & HYPERTENSJON_ALENE ==0 & GESDM==0 || FAR_LOPEN

> R: , or

Fitting fixed-effects model:

Iteration 0: Log likelihood = -52014.817

Iteration 1: Log likelihood = -23149.431

Iteration 2: Log likelihood = -22827.54

Iteration 3: Log likelihood = -22764.024

Iteration 4: Log likelihood = -22763.904

Iteration 5: Log likelihood = -22763.904

Refining starting values:

Grid node 0: Log likelihood = -23198.959

Fitting full model:

Iteration 0: Log likelihood = -23198.959

Iteration 1: Log likelihood = -22671.007

Iteration 2: Log likelihood = -22657.601

Iteration 3: Log likelihood = -22657.583

Iteration 4: Log likelihood = -22657.583

Mixed-effects logistic regression Number of obs = 486,785

Group variable: FAR_LOPENR Number of groups = 215,573

Obs per group:

min = 1

avg = 2.3

max = 15

Integration method: mvaghermite Integration pts. = 7

Wald chi2(13) = 342.20

Log likelihood = -22657.583 Prob > chi2 = 0.0000

--------------------------------------------------------------------------------------

fetalloss_total | Odds ratio Std. err. z P>|z| [95% conf. interval]

---------------------+----------------------------------------------------------------

fam_chd60_final_f_v2 | 1.010445 .0524978 0.20 0.841 .9126169 1.11876

|

FAAR_K4 |

1978_1988 | .6804038 .029125 -9.00 0.000 .6256489 .7399507

1989_1999 | .6741537 .0390919 -6.80 0.000 .6017285 .755296

2000_max | .4736351 .04758 -7.44 0.000 .3889867 .5767041

|

fAge_KAT_K4 |

min-25 | .9197506 .0493586 -1.56 0.119 .8279234 1.021763

31-39 | 1.060641 .054319 1.15 0.250 .9593463 1.17263

40-max | 1.183888 .0969241 2.06 0.039 1.008378 1.389946

|

MORS_ALDER_K5 |

min_19 | .9907376 .0751819 -0.12 0.902 .8538182 1.149614

25-29 | .9697712 .048736 -0.61 0.541 .8788039 1.070155

30-34 | 1.140339 .0736171 2.03 0.042 1.004807 1.294151

35-max | 1.805505 .1380084 7.73 0.000 1.5543 2.097309

|

mCivil |

unmarried | .4340963 .0392666 -9.23 0.000 .3635715 .5183014

wdo_divo_sep | .8059388 .1427664 -1.22 0.223 .5695315 1.140477

|

_cons | .004816 .0003398 -75.63 0.000 .0041941 .0055302

---------------------+----------------------------------------------------------------

FAR_LOPENR |

var(_cons)| 1.494666 .1074977 1.298151 1.720931

--------------------------------------------------------------------------------------

Note: Estimates are transformed only in the first equation to odds ratios.

Note: _cons estimates baseline odds (conditional on zero random effects).

LR test vs. logistic model: chibar2(01) = 212.64 Prob >= chibar2 = 0.0000

Regression S2.8. Paternal family history of premature CHD and neonatal death (excluding mother with risk factors of perinatal death)

. melogit neo fam_chd60_final_f_v2 i.FAAR_K4 i.fAge_KAT_K4 i.MORS_ALDER_K5 i.mCivil if preeclampsia_ext==0 & HYPERTENSJON_KRONISK ==0 & HYPERTENSJON_ALENE ==0 & GESDM==0 || FAR_LOPENR: , or

Fitting fixed-effects model:

Iteration 0: Log likelihood = -46931.057

Iteration 1: Log likelihood = -14182.157

Iteration 2: Log likelihood = -14065.611

Iteration 3: Log likelihood = -14059.209

Iteration 4: Log likelihood = -14059.185

Iteration 5: Log likelihood = -14059.185

Refining starting values:

Grid node 0: Log likelihood = -14322.082

Fitting full model:

Iteration 0: Log likelihood = -14322.082

Iteration 1: Log likelihood = -14004.067

Iteration 2: Log likelihood = -13997.173

Iteration 3: Log likelihood = -13996.972

Iteration 4: Log likelihood = -13996.972

Mixed-effects logistic regression Number of obs = 482,835

Group variable: FAR_LOPENR Number of groups = 215,315

Obs per group:

min = 1

avg = 2.2

max = 15

Integration method: mvaghermite Integration pts. = 7

Wald chi2(13) = 365.58

Log likelihood = -13996.972 Prob > chi2 = 0.0000

--------------------------------------------------------------------------------------

neo | Odds ratio Std. err. z P>|z| [95% conf. interval]

---------------------+----------------------------------------------------------------

fam_chd60_final_f_v2 | .9862695 .0684982 -0.20 0.842 .8607521 1.13009

|

FAAR_K4 |

1978_1988 | .5752638 .0314491 -10.11 0.000 .5168122 .6403264

1989_1999 | .4204083 .0363348 -10.03 0.000 .3548985 .4980103

2000_max | .2304446 .0428894 -7.89 0.000 .160009 .3318858

|

fAge_KAT_K4 |

min-25 | 1.045535 .0680987 0.68 0.494 .920232 1.1879

31-39 | 1.076592 .0757611 1.05 0.294 .9378877 1.235808

40-max | 1.201201 .1546116 1.42 0.154 .9333707 1.545886

|

MORS_ALDER_K5 |

min_19 | 1.509211 .1142166 5.44 0.000 1.301162 1.750527

25-29 | .8969713 .0573316 -1.70 0.089 .791357 1.016681

30-34 | .9121814 .0806681 -1.04 0.299 .7670186 1.084817

35-max | 1.079321 .1256942 0.66 0.512 .8590581 1.35606

|

mCivil |

unmarried | .664167 .0595719 -4.56 0.000 .5570953 .7918176

wdo_divo_sep | .9021437 .2185662 -0.43 0.671 .5611146 1.450441

|

_cons | .0027237 .0002753 -58.42 0.000 .0022342 .0033206

---------------------+----------------------------------------------------------------

FAR_LOPENR |

var(_cons)| 1.839242 .1688946 1.536295 2.201929

--------------------------------------------------------------------------------------

Note: Estimates are transformed only in the first equation to odds ratios.

Note: _cons estimates baseline odds (conditional on zero random effects).

LR test vs. logistic model: chibar2(01) = 124.43 Prob >= chibar2 = 0.0000

Regression S2.9. Paternal family history of premature CHD and perinatal death (excluding mother with risk factors of perinatal death)

. melogit totalloss_12aug fam_chd60_final_f_v2 i.FAAR_K4 i.fAge_KAT_K4 i.MORS_ALDER_K5 i.mCivil if preeclampsia_ext==0 & HYPERTENSJON_KRONISK ==0 & HYPERTENSJON_ALENE ==0 & GESDM==0|| FAR_LOPENR:

> , or

Fitting fixed-effects model:

Iteration 0: Log likelihood = -58132.358

Iteration 1: Log likelihood = -33197.919

Iteration 2: Log likelihood = -32891.477

Iteration 3: Log likelihood = -32888.199

Iteration 4: Log likelihood = -32888.193

Iteration 5: Log likelihood = -32888.193

Refining starting values:

Grid node 0: Log likelihood = -33489.845

Fitting full model:

Iteration 0: Log likelihood = -33489.845

Iteration 1: Log likelihood = -32727.808

Iteration 2: Log likelihood = -32684.959

Iteration 3: Log likelihood = -32684.82

Iteration 4: Log likelihood = -32684.82

Mixed-effects logistic regression Number of obs = 486,785

Group variable: FAR_LOPENR Number of groups = 215,573

Obs per group:

min = 1

avg = 2.3

max = 15

Integration method: mvaghermite Integration pts. = 7

Wald chi2(13) = 493.34

Log likelihood = -32684.82 Prob > chi2 = 0.0000

--------------------------------------------------------------------------------------

totalloss_12aug | Odds ratio Std. err. z P>|z| [95% conf. interval]

---------------------+----------------------------------------------------------------

fam_chd60_final_f_v2 | 1.002005 .0425195 0.05 0.962 .9220402 1.088906

|

FAAR_K4 |

1978_1988 | .634456 .0216889 -13.31 0.000 .5933395 .6784219

1989_1999 | .5769333 .0279963 -11.33 0.000 .5245901 .6344992

2000_max | .3848353 .0340852 -10.78 0.000 .3235066 .4577904

|

fAge_KAT_K4 |

min-25 | .9735794 .0408185 -0.64 0.523 .8967755 1.056961

31-39 | 1.065117 .0446462 1.51 0.132 .9811104 1.156317

40-max | 1.189548 .0829895 2.49 0.013 1.037522 1.36385

|

MORS_ALDER_K5 |

min_19 | 1.225676 .0661991 3.77 0.000 1.10256 1.36254

25-29 | .9411628 .0376389 -1.52 0.129 .8702091 1.017902

30-34 | 1.057107 .0557075 1.05 0.292 .9533713 1.172129

35-max | 1.556005 .0999497 6.88 0.000 1.371938 1.764769

|

mCivil |

unmarried | .5253455 .0336183 -10.06 0.000 .4634194 .5955465

wdo_divo_sep | .8364721 .1209781 -1.23 0.217 .6300037 1.110606

|

_cons | .0084182 .0004446 -90.45 0.000 .0075903 .0093363

---------------------+----------------------------------------------------------------

FAR_LOPENR |

var(_cons)| 1.423848 .0777383 1.279352 1.584663

--------------------------------------------------------------------------------------

Note: Estimates are transformed only in the first equation to odds ratios.

Note: _cons estimates baseline odds (conditional on zero random effects).

LR test vs. logistic model: chibar2(01) = 406.74 Prob >= chibar2 = 0.0000

**Family History of Stroke**

Regression S2.10. Paternal family history of premature stroke and stillbirth (excluding mother with risk factors of perinatal death)

melogit fetalloss_total fam_stroke70_final_f_v2 i.FAAR_K4 i.fAge_KAT_K4 i.MORS_ALDER_K5 i.mCivil if preeclampsia_ext==0 & HYPERTENSJON_KRONISK ==0 & HYPERTENSJON_ALENE ==0 & GESDM==0|| FAR_LOPE

> NR: , or

Fitting fixed-effects model:

Iteration 0: Log likelihood = -19792.037

Iteration 1: Log likelihood = -7747.9767

Iteration 2: Log likelihood = -7676.7322

Iteration 3: Log likelihood = -7664.8855

Iteration 4: Log likelihood = -7664.8727

Iteration 5: Log likelihood = -7664.8727

Refining starting values:

Grid node 0: Log likelihood = -7799.3584

Fitting full model:

Iteration 0: Log likelihood = -7799.3584

Iteration 1: Log likelihood = -7613.4641

Iteration 2: Log likelihood = -7612.4446

Iteration 3: Log likelihood = -7612.4437

Iteration 4: Log likelihood = -7612.4437

Mixed-effects logistic regression Number of obs = 192,230

Group variable: FAR_LOPENR Number of groups = 83,375

Obs per group:

min = 1

avg = 2.3

max = 13

Integration method: mvaghermite Integration pts. = 7

Wald chi2(13) = 136.50

Log likelihood = -7612.4437 Prob > chi2 = 0.0000

-----------------------------------------------------------------------------------------

fetalloss_total | Odds ratio Std. err. z P>|z| [95% conf. interval]

------------------------+----------------------------------------------------------------

fam_stroke70_final_f_v2 | .9820705 .1011827 -0.18 0.861 .802497 1.201827

|

FAAR_K4 |

1978_1988 | .7593944 .1003143 -2.08 0.037 .5861727 .9838055

1989_1999 | .8299203 .1264096 -1.22 0.221 .615723 1.118632

2000_max | .6034663 .1093312 -2.79 0.005 .4230962 .8607301

|

fAge_KAT_K4 |

min-25 | .9597475 .1029792 -0.38 0.702 .7777224 1.184375

31-39 | 1.073044 .1020178 0.74 0.458 .8906166 1.292837

40-max | .9705508 .1402946 -0.21 0.836 .7310983 1.28843

|

MORS_ALDER_K5 |

min_19 | .9943603 .1531575 -0.04 0.971 .7352524 1.344779

25-29 | .8348173 .0792131 -1.90 0.057 .6931444 1.005447

30-34 | 1.125559 .126622 1.05 0.293 .9028393 1.40322

35-max | 1.846935 .2384996 4.75 0.000 1.433949 2.378864

|

mCivil |

unmarried | .4844088 .0680898 -5.16 0.000 .3677603 .6380565

wdo_divo_sep | 1.244872 .3175947 0.86 0.391 .75503 2.05251

|

_cons | .0034184 .0006037 -32.16 0.000 .0024183 .0048321

------------------------+----------------------------------------------------------------

FAR_LOPENR |

var(_cons)| 1.81094 .1932975 1.469088 2.232339

-----------------------------------------------------------------------------------------

Note: Estimates are transformed only in the first equation to odds ratios.

Note: _cons estimates baseline odds (conditional on zero random effects).

LR test vs. logistic model: chibar2(01) = 104.86 Prob >= chibar2 = 0.0000

Regression S2.11. Paternal family history of premature stroke and neonatal death (excluding mother with risk factors of perinatal death)

. melogit neo fam_stroke70_final_f_v2 i.FAAR_K4 i.fAge_KAT_K4 i.MORS_ALDER_K5 i.mCivil if preeclampsia_ext==0 & HYPERTENSJON_KRONISK ==0 & HYPERTENSJON_ALENE ==0 & GESDM==0|| FAR_LOPENR: , or

Fitting fixed-effects model:

Iteration 0: Log likelihood = -17881.472

Iteration 1: Log likelihood = -4305.9139

Iteration 2: Log likelihood = -4251.3658

Iteration 3: Log likelihood = -4249.5345

Iteration 4: Log likelihood = -4249.5301

Iteration 5: Log likelihood = -4249.5301

Refining starting values:

Grid node 0: Log likelihood = -4326.6796

Fitting full model:

Iteration 0: Log likelihood = -4326.6796

Iteration 1: Log likelihood = -4234.0122

Iteration 2: Log likelihood = -4231.3126

Iteration 3: Log likelihood = -4230.5145

Iteration 4: Log likelihood = -4230.5128

Iteration 5: Log likelihood = -4230.5128

Mixed-effects logistic regression Number of obs = 190,941

Group variable: FAR_LOPENR Number of groups = 83,308

Obs per group:

min = 1

avg = 2.3

max = 12

Integration method: mvaghermite Integration pts. = 7

Wald chi2(13) = 88.33

Log likelihood = -4230.5128 Prob > chi2 = 0.0000

-----------------------------------------------------------------------------------------

neo | Odds ratio Std. err. z P>|z| [95% conf. interval]

------------------------+----------------------------------------------------------------

fam_stroke70_final_f_v2 | 1.094961 .1487082 0.67 0.504 .8390652 1.428899

|

FAAR_K4 |

1978_1988 | .6116125 .0874566 -3.44 0.001 .4621256 .8094549

1989_1999 | .4793429 .0878549 -4.01 0.000 .3346851 .6865249

2000_max | .2932552 .076639 -4.69 0.000 .1757088 .4894382

|

fAge_KAT_K4 |

min-25 | 1.045861 .1383012 0.34 0.735 .8070746 1.355295

31-39 | .9578251 .1285702 -0.32 0.748 .736255 1.246075

40-max | 1.075652 .2546676 0.31 0.758 .6763079 1.710801

|

MORS_ALDER_K5 |

min_19 | 1.500714 .2338801 2.60 0.009 1.105711 2.036827

25-29 | .9180698 .1120133 -0.70 0.484 .7228047 1.166086

30-34 | .9076384 .1454128 -0.60 0.545 .6630432 1.242464

35-max | .9914965 .2081894 -0.04 0.968 .6569922 1.496312

|

mCivil |

unmarried | .6538625 .0974811 -2.85 0.004 .4881858 .8757654

wdo_divo_sep | .6637488 .3385124 -0.80 0.422 .2442814 1.803504

|

_cons | .0022625 .0005303 -25.99 0.000 .0014292 .0035817

------------------------+----------------------------------------------------------------

FAR_LOPENR |

var(_cons)| 2.034427 .3339841 1.4747 2.8066

-----------------------------------------------------------------------------------------

Note: Estimates are transformed only in the first equation to odds ratios.

Note: _cons estimates baseline odds (conditional on zero random effects).

LR test vs. logistic model: chibar2(01) = 38.03 Prob >= chibar2 = 0.0000

Regression S2.12. Paternal family history of premature stroke and perinatal death (excluding mother with risk factors of perinatal death)

. melogit totalloss_12aug fam_stroke70_final_f_v2 i.FAAR_K4 i.fAge_KAT_K4 i.MORS_ALDER_K5 i.mCivil if preeclampsia_ext==0 & HYPERTENSJON_KRONISK ==0 & HYPERTENSJON_ALENE ==0 & GESDM==0|| FAR_LOPE

> NR: , or

Fitting fixed-effects model:

Iteration 0: Log likelihood = -21558.971

Iteration 1: Log likelihood = -10950.175

Iteration 2: Log likelihood = -10738.77

Iteration 3: Log likelihood = -10736.799

Iteration 4: Log likelihood = -10736.798

Iteration 5: Log likelihood = -10736.798

Refining starting values:

Grid node 0: Log likelihood = -10914.538

Fitting full model:

Iteration 0: Log likelihood = -10914.538

Iteration 1: Log likelihood = -10643.478

Iteration 2: Log likelihood = -10642.68

Iteration 3: Log likelihood = -10642.68

Mixed-effects logistic regression Number of obs = 192,230

Group variable: FAR_LOPENR Number of groups = 83,375

Obs per group:

min = 1

avg = 2.3

max = 13

Integration method: mvaghermite Integration pts. = 7

Wald chi2(13) = 122.09

Log likelihood = -10642.68 Prob > chi2 = 0.0000

-----------------------------------------------------------------------------------------

totalloss_12aug | Odds ratio Std. err. z P>|z| [95% conf. interval]

------------------------+----------------------------------------------------------------

fam_stroke70_final_f_v2 | 1.010333 .0852704 0.12 0.903 .8562973 1.192078

|

FAAR_K4 |

1978_1988 | .6829923 .0672715 -3.87 0.000 .5630882 .8284287

1989_1999 | .6734276 .0791779 -3.36 0.001 .5348245 .8479506

2000_max | .4717082 .0692965 -5.11 0.000 .3536937 .6290999

|

fAge_KAT_K4 |

min-25 | .9974825 .0841761 -0.03 0.976 .8454221 1.176893

31-39 | 1.033489 .0810882 0.42 0.675 .8861762 1.20529

40-max | .9797225 .1219879 -0.16 0.869 .7675695 1.250514

|

MORS_ALDER_K5 |

min_19 | 1.225969 .1354713 1.84 0.065 .9872353 1.522434

25-29 | .8642795 .0656805 -1.92 0.055 .7446764 1.003092

30-34 | 1.059952 .0986743 0.63 0.532 .8831718 1.272118

35-max | 1.603881 .1765502 4.29 0.000 1.292631 1.990076

|

mCivil |

unmarried | .5492565 .0567001 -5.80 0.000 .4486474 .6724271

wdo_divo_sep | 1.049704 .2424326 0.21 0.834 .6675427 1.650649

|

_cons | .0062235 .0008295 -38.11 0.000 .0047928 .0080813

------------------------+----------------------------------------------------------------

FAR_LOPENR |

var(_cons)| 1.766882 .1467683 1.501418 2.079284

-----------------------------------------------------------------------------------------

Note: Estimates are transformed only in the first equation to odds ratios.

Note: _cons estimates baseline odds (conditional on zero random effects).

LR test vs. logistic model: chibar2(01) = 188.24 Prob >= chibar2 = 0.0000

.

end of do-file

Additional analysis(population with information on paternal education)

**Family history of CHD**

Regression S2.13. Paternal family history of premature CHD and stillbirth (subpopulation where paternal education is available and adjusted for education)

. melogit fetalloss_total fam_chd60_final_f_v2 i.FAAR_K4 i.fAge_KAT_K4 i.MORS_ALDER_K5 ib3.education_f if education_f!=. || FAR_LOPENR: , or

Fitting fixed-effects model:

Iteration 0: Log likelihood = -7018.3879

Iteration 1: Log likelihood = -2824.2263

Iteration 2: Log likelihood = -2794.3568

Iteration 3: Log likelihood = -2788.4136

Iteration 4: Log likelihood = -2788.41

Iteration 5: Log likelihood = -2788.41

Refining starting values:

Grid node 0: Log likelihood = -2838.8535

Fitting full model:

Iteration 0: Log likelihood = -2838.8535

Iteration 1: Log likelihood = -2771.8985

Iteration 2: Log likelihood = -2771.6429

Iteration 3: Log likelihood = -2771.6428

Mixed-effects logistic regression Number of obs = 67,648

Group variable: FAR_LOPENR Number of groups = 28,720

Obs per group:

min = 1

avg = 2.4

max = 10

Integration method: mvaghermite Integration pts. = 7

Wald chi2(13) = 58.53

Log likelihood = -2771.6428 Prob > chi2 = 0.0000

--------------------------------------------------------------------------------------

fetalloss_total | Odds ratio Std. err. z P>|z| [95% conf. interval]

---------------------+----------------------------------------------------------------

fam_chd60_final_f_v2 | .8217897 .1201111 -1.34 0.179 .6170932 1.094386

|

FAAR_K4 |

1978_1988 | .5547492 .1024576 -3.19 0.001 .3862664 .7967215

1989_1999 | .4350472 .0907038 -3.99 0.000 .2891131 .6546438

2000_max | .2979808 .0724607 -4.98 0.000 .1850119 .4799289

|

fAge_KAT_K4 |

min-25 | .8031994 .1401075 -1.26 0.209 .5706142 1.130588

31-39 | 1.25638 .1830706 1.57 0.117 .9442564 1.671675

40-max | 1.019482 .2324109 0.08 0.933 .6521259 1.593777

|

MORS_ALDER_K5 |

min_19 | .8746931 .2086158 -0.56 0.575 .5480783 1.395947

25-29 | .9158748 .1443924 -0.56 0.577 .6724187 1.247477

30-34 | 1.277635 .2383484 1.31 0.189 .8863625 1.841629

35-max | 2.131754 .4550829 3.55 0.000 1.402892 3.239292

|

education_f |

primary | 1.26942 .1819994 1.66 0.096 .9584444 1.681295

secondary | .9610062 .1062791 -0.36 0.719 .7737319 1.193608

|

_cons | .0052462 .0013918 -19.79 0.000 .003119 .0088241

---------------------+----------------------------------------------------------------

FAR_LOPENR |

var(_cons)| 1.681844 .3123361 1.168715 2.420264

--------------------------------------------------------------------------------------

Note: Estimates are transformed only in the first equation to odds ratios.

Note: _cons estimates baseline odds (conditional on zero random effects).

LR test vs. logistic model: chibar2(01) = 33.53 Prob >= chibar2 = 0.0000

Regression S2.14. Paternal family history of premature CHD and neonatal death (subpopulation where paternal education is available and adjusted for education)

. melogit neo fam_chd60_final_f_v2 i.FAAR_K4 i.fAge_KAT_K4 i.MORS_ALDER_K5 ib3.education_f if education_f!=. || FAR_LOPENR: , or

Fitting fixed-effects model:

Iteration 0: Log likelihood = -6180.2659

Iteration 1: Log likelihood = -1298.339

Iteration 2: Log likelihood = -1262.7104

Iteration 3: Log likelihood = -1262.1201

Iteration 4: Log likelihood = -1262.1172

Iteration 5: Log likelihood = -1262.1172

Refining starting values:

Grid node 0: Log likelihood = -1287.2551

Fitting full model:

Iteration 0: Log likelihood = -1287.2551

Iteration 1: Log likelihood = -1269.7244

Iteration 2: Log likelihood = -1260.8935

Iteration 3: Log likelihood = -1260.6357

Iteration 4: Log likelihood = -1260.6352

Iteration 5: Log likelihood = -1260.6352

Mixed-effects logistic regression Number of obs = 67,175

Group variable: FAR_LOPENR Number of groups = 28,695

Obs per group:

min = 1

avg = 2.3

max = 10

Integration method: mvaghermite Integration pts. = 7

Wald chi2(13) = 35.38

Log likelihood = -1260.6352 Prob > chi2 = 0.0007

--------------------------------------------------------------------------------------

neo | Odds ratio Std. err. z P>|z| [95% conf. interval]

---------------------+----------------------------------------------------------------

fam_chd60_final_f_v2 | .9594951 .2010204 -0.20 0.844 .6363713 1.446688

|

FAAR_K4 |

1978_1988 | .7218771 .1742152 -1.35 0.177 .4498185 1.158482

1989_1999 | .4250622 .1235292 -2.94 0.003 .2404812 .7513183

2000_max | .2594791 .1001533 -3.50 0.000 .1217747 .5529016

|

fAge_KAT_K4 |

min-25 | 1.089502 .247693 0.38 0.706 .697769 1.701158

31-39 | .984806 .2250064 -0.07 0.947 .6293176 1.541102

40-max | 1.314702 .5214879 0.69 0.490 .6042145 2.860644

|

MORS_ALDER_K5 |

min_19 | .9206538 .2734249 -0.28 0.781 .5143965 1.647763

25-29 | 1.010835 .2201238 0.05 0.961 .6596585 1.548965

30-34 | 1.08412 .3105888 0.28 0.778 .6183258 1.900805

35-max | .9963098 .3863153 -0.01 0.992 .4659554 2.130318

|

education_f |

primary | 1.682197 .3786658 2.31 0.021 1.082106 2.615074

secondary | 1.422543 .2609718 1.92 0.055 .9929073 2.038084

|

_cons | .0019363 .0008964 -13.49 0.000 .0007815 .0047975

---------------------+----------------------------------------------------------------

FAR_LOPENR |

var(_cons)| 1.272697 .6839888 .443876 3.649122

--------------------------------------------------------------------------------------

Note: Estimates are transformed only in the first equation to odds ratios.

Note: _cons estimates baseline odds (conditional on zero random effects).

LR test vs. logistic model: chibar2(01) = 2.96 Prob >= chibar2 = 0.0426

Regression S2.15. Paternal family history of premature CHD and perinatal death (subpopulation where paternal education is available and adjused for education)

. melogit totalloss_12aug fam_chd60_final_f_v2 i.FAAR_K4 i.fAge_KAT_K4 ib3.education_f if education_f!=.|| FAR_LOPENR: , or

Fitting fixed-effects model:

Iteration 0: Log likelihood = -7531.3887

Iteration 1: Log likelihood = -3765.9811

Iteration 2: Log likelihood = -3685.5553

Iteration 3: Log likelihood = -3684.9752

Iteration 4: Log likelihood = -3684.9748

Iteration 5: Log likelihood = -3684.9748

Refining starting values:

Grid node 0: Log likelihood = -3751.4531

Fitting full model:

Iteration 0: Log likelihood = -3751.4531

Iteration 1: Log likelihood = -3662.6656

Iteration 2: Log likelihood = -3661.2388

Iteration 3: Log likelihood = -3661.2322

Iteration 4: Log likelihood = -3661.2322

Mixed-effects logistic regression Number of obs = 67,648

Group variable: FAR_LOPENR Number of groups = 28,720

Obs per group:

min = 1

avg = 2.4

max = 10

Integration method: mvaghermite Integration pts. = 7

Wald chi2(9) = 43.33

Log likelihood = -3661.2322 Prob > chi2 = 0.0000

--------------------------------------------------------------------------------------

totalloss_12aug | Odds ratio Std. err. z P>|z| [95% conf. interval]

---------------------+----------------------------------------------------------------

fam_chd60_final_f_v2 | .8678875 .1060586 -1.16 0.246 .6830365 1.102765

|

FAAR_K4 |

1978_1988 | .6105309 .0896061 -3.36 0.001 .4579086 .8140225

1989_1999 | .4679203 .0781958 -4.54 0.000 .3372288 .6492607

2000_max | .3294571 .0662114 -5.52 0.000 .2221937 .4885015

|

fAge_KAT_K4 |

min-25 | .8994889 .1119136 -0.85 0.395 .7048393 1.147893

31-39 | 1.380728 .1502166 2.97 0.003 1.115581 1.708892

40-max | 1.512427 .2662762 2.35 0.019 1.071057 2.135679

|

education_f |

primary | 1.299653 .1578026 2.16 0.031 1.024414 1.648844

secondary | 1.021978 .0967299 0.23 0.818 .8489369 1.23029

|

_cons | .0072483 .0014534 -24.57 0.000 .0048929 .0107378

---------------------+----------------------------------------------------------------

FAR_LOPENR |

var(_cons)| 1.559221 .2467628 1.143395 2.126273

--------------------------------------------------------------------------------------

Note: Estimates are transformed only in the first equation to odds ratios.

Note: _cons estimates baseline odds (conditional on zero random effects).

LR test vs. logistic model: chibar2(01) = 47.49 Prob >= chibar2 = 0.0000

Regression S2.16. Paternal family history of premature CHD and stillbirth (subpopulation where paternal education is available and adjusted for marital status)

. melogit fetalloss_total fam_chd60_final_f_v2 i.FAAR_K4 i.fAge_KAT_K4 i.MORS_ALDER_K5 i.mCivil if education_f!=. || FAR_LOPENR: , or

Fitting fixed-effects model:

Iteration 0: Log likelihood = -7018.377

Iteration 1: Log likelihood = -2824.1145

Iteration 2: Log likelihood = -2793.743

Iteration 3: Log likelihood = -2787.8986

Iteration 4: Log likelihood = -2787.8941

Iteration 5: Log likelihood = -2787.8941

Refining starting values:

Grid node 0: Log likelihood = -2838.2313

Fitting full model:

Iteration 0: Log likelihood = -2838.2313

Iteration 1: Log likelihood = -2771.1853

Iteration 2: Log likelihood = -2770.9369

Iteration 3: Log likelihood = -2770.9368

Mixed-effects logistic regression Number of obs = 67,648

Group variable: FAR_LOPENR Number of groups = 28,720

Obs per group:

min = 1

avg = 2.4

max = 10

Integration method: mvaghermite Integration pts. = 7

Wald chi2(13) = 59.33

Log likelihood = -2770.9368 Prob > chi2 = 0.0000

--------------------------------------------------------------------------------------

fetalloss_total | Odds ratio Std. err. z P>|z| [95% conf. interval]

---------------------+----------------------------------------------------------------

fam_chd60_final_f_v2 | .8322423 .1216605 -1.26 0.209 .6249104 1.108362

|

FAAR_K4 |

1978_1988 | .5535888 .1026254 -3.19 0.001 .3849372 .7961314

1989_1999 | .4211959 .0875462 -4.16 0.000 .2802598 .6330056

2000_max | .2835784 .068595 -5.21 0.000 .1765125 .4555866

|

fAge_KAT_K4 |

min-25 | .8154574 .1429144 -1.16 0.244 .5783922 1.149688

31-39 | 1.278279 .1863022 1.68 0.092 .9606558 1.700919

40-max | 1.057623 .2410854 0.25 0.806 .6765487 1.653341

|

MORS_ALDER_K5 |

min_19 | 1.011962 .2482072 0.05 0.961 .62573 1.636595

25-29 | .874074 .1377946 -0.85 0.393 .6417404 1.190521

30-34 | 1.201684 .2230626 0.99 0.322 .8351914 1.728999

35-max | 2.009911 .425994 3.29 0.001 1.326684 3.044992

|

mCivil |

unmarried | .6568803 .1338761 -2.06 0.039 .4405613 .9794136

wdo_divo_sep | 1.459277 .5829444 0.95 0.344 .6669666 3.192797

|

_cons | .0056923 .0014555 -20.21 0.000 .0034486 .009396

---------------------+----------------------------------------------------------------

FAR_LOPENR |

var(_cons)| 1.693514 .3128057 1.179139 2.432274

--------------------------------------------------------------------------------------

Note: Estimates are transformed only in the first equation to odds ratios.

Note: _cons estimates baseline odds (conditional on zero random effects).

LR test vs. logistic model: chibar2(01) = 33.91 Prob >= chibar2 = 0.0000

Regression S2.17. Paternal family history of premature CHD and neonatal death (subpopulation where paternal education is available and adjusted for marital status)

. melogit neo fam_chd60_final_f_v2 i.FAAR_K4 i.fAge_KAT_K4 i.MORS_ALDER_K5 i.mCivil if education_f!=. || FAR_LOPENR: , or

Fitting fixed-effects model:

Iteration 0: Log likelihood = -6180.3307

Iteration 1: Log likelihood = -1298.9578

Iteration 2: Log likelihood = -1265.5909

Iteration 3: Log likelihood = -1265.0734

Iteration 4: Log likelihood = -1265.0703

Iteration 5: Log likelihood = -1265.0703

Refining starting values:

Grid node 0: Log likelihood = -1290.1875

Fitting full model:

Iteration 0: Log likelihood = -1290.1875

Iteration 1: Log likelihood = -1264.9087

Iteration 2: Log likelihood = -1264.0575

Iteration 3: Log likelihood = -1263.5168

Iteration 4: Log likelihood = -1263.5142

Iteration 5: Log likelihood = -1263.5142

Mixed-effects logistic regression Number of obs = 67,175

Group variable: FAR_LOPENR Number of groups = 28,695

Obs per group:

min = 1

avg = 2.3

max = 10

Integration method: mvaghermite Integration pts. = 7

Wald chi2(13) = 29.88

Log likelihood = -1263.5142 Prob > chi2 = 0.0049

--------------------------------------------------------------------------------------

neo | Odds ratio Std. err. z P>|z| [95% conf. interval]

---------------------+----------------------------------------------------------------

fam_chd60_final_f_v2 | .9727931 .2036849 -0.13 0.895 .645349 1.466379

|

FAAR_K4 |

1978_1988 | .7215632 .1743852 -1.35 0.177 .4493229 1.158751

1989_1999 | .4271416 .12367 -2.94 0.003 .2421721 .7533896

2000_max | .2580699 .0992384 -3.52 0.000 .1214551 .5483513

|

fAge_KAT_K4 |

min-25 | 1.100613 .2516411 0.42 0.675 .7031019 1.722864

31-39 | .9907674 .2271851 -0.04 0.968 .6321052 1.552938

40-max | 1.351368 .5368767 0.76 0.448 .6203049 2.94403

|

MORS_ALDER_K5 |

min_19 | .9505023 .2912537 -0.17 0.868 .5213474 1.732922

25-29 | .9616324 .2099135 -0.18 0.858 .6269045 1.475084

30-34 | .9894799 .2831673 -0.04 0.971 .5646923 1.733812

35-max | .8925835 .34556 -0.29 0.769 .4179359 1.906286

|

mCivil |

unmarried | 1.018303 .2458776 0.08 0.940 .6343768 1.634582

wdo_divo_sep | .5957378 .6018266 -0.51 0.608 .0822534 4.314759

|

_cons | .0026176 .0011537 -13.49 0.000 .0011034 .0062096

---------------------+----------------------------------------------------------------

FAR_LOPENR |

var(_cons)| 1.297584 .6783952 .4657097 3.615394

--------------------------------------------------------------------------------------

Note: Estimates are transformed only in the first equation to odds ratios.

Note: _cons estimates baseline odds (conditional on zero random effects).

LR test vs. logistic model: chibar2(01) = 3.11 Prob >= chibar2 = 0.0389

Regression S2.18. Paternal family history of premature CHD and perinatal death (subpopulation where paternal education is available and adjusted for marital status)

. melogit totalloss_12aug fam_chd60_final_f_v2 i.FAAR_K4 i.fAge_KAT_K4 i.mCivil if education_f!=.|| FAR_LOPENR: , or

Fitting fixed-effects model:

Iteration 0: Log likelihood = -7531.5001

Iteration 1: Log likelihood = -3766.5929

Iteration 2: Log likelihood = -3686.7606

Iteration 3: Log likelihood = -3686.1475

Iteration 4: Log likelihood = -3686.1471

Iteration 5: Log likelihood = -3686.1471

Refining starting values:

Grid node 0: Log likelihood = -3752.5872

Fitting full model:

Iteration 0: Log likelihood = -3752.5872

Iteration 1: Log likelihood = -3663.5281

Iteration 2: Log likelihood = -3662.2126

Iteration 3: Log likelihood = -3662.2059

Iteration 4: Log likelihood = -3662.2059

Mixed-effects logistic regression Number of obs = 67,648

Group variable: FAR_LOPENR Number of groups = 28,720

Obs per group:

min = 1

avg = 2.4

max = 10

Integration method: mvaghermite Integration pts. = 7

Wald chi2(9) = 41.09

Log likelihood = -3662.2059 Prob > chi2 = 0.0000

--------------------------------------------------------------------------------------

totalloss_12aug | Odds ratio Std. err. z P>|z| [95% conf. interval]

---------------------+----------------------------------------------------------------

fam_chd60_final_f_v2 | .87775 .1072347 -1.07 0.286 .6908433 1.115224

|

FAAR_K4 |

1978_1988 | .5969836 .0875466 -3.52 0.000 .4478526 .7957739

1989_1999 | .4459407 .0742418 -4.85 0.000 .3217852 .6179996

2000_max | .3083705 .0616056 -5.89 0.000 .2084594 .4561673

|

fAge_KAT_K4 |

min-25 | .9443433 .1188327 -0.46 0.649 .7379345 1.208487

31-39 | 1.376792 .1492717 2.95 0.003 1.113221 1.702769

40-max | 1.529269 .2683424 2.42 0.015 1.084233 2.156975

|

mCivil |

unmarried | .7819452 .1185297 -1.62 0.105 .580963 1.052456

wdo_divo_sep | 1.288486 .4797729 0.68 0.496 .6210572 2.673179

|

_cons | .0079806 .0015302 -25.19 0.000 .0054806 .011621

---------------------+----------------------------------------------------------------

FAR_LOPENR |

var(_cons)| 1.565199 .2466067 1.149364 2.131481

--------------------------------------------------------------------------------------

Note: Estimates are transformed only in the first equation to odds ratios.

Note: _cons estimates baseline odds (conditional on zero random effects).

LR test vs. logistic model: chibar2(01) = 47.88 Prob >= chibar2 = 0.0000

.

end of do-file

**Family History of Stroke**

Regression S2.19. Paternal family history of premature stroke and stillbirth (subpopulation where paternal education is available and adjusted for education)

melogit fetalloss_total fam_stroke70_final_f_v2 i.FAAR_K4 i.fAge_KAT_K4 i.MORS_ALDER_K5 ib3.education_f if education_f!=. || FAR_LOPENR: , or

Fitting fixed-effects model:

Iteration 0: Log likelihood = -7018.4389

Iteration 1: Log likelihood = -2824.6857

Iteration 2: Log likelihood = -2795.1102

Iteration 3: Log likelihood = -2789.3292

Iteration 4: Log likelihood = -2789.3256

Iteration 5: Log likelihood = -2789.3256

Refining starting values:

Grid node 0: Log likelihood = -2839.8144

Fitting full model:

Iteration 0: Log likelihood = -2839.8144

Iteration 1: Log likelihood = -2772.838

Iteration 2: Log likelihood = -2772.5813

Iteration 3: Log likelihood = -2772.5812

Mixed-effects logistic regression Number of obs = 67,648

Group variable: FAR_LOPENR Number of groups = 28,720

Obs per group:

min = 1

avg = 2.4

max = 10

Integration method: mvaghermite Integration pts. = 7

Wald chi2(13) = 56.69

Log likelihood = -2772.5812 Prob > chi2 = 0.0000

-----------------------------------------------------------------------------------------

fetalloss_total | Odds ratio Std. err. z P>|z| [95% conf. interval]

------------------------+----------------------------------------------------------------

fam_stroke70_final_f_v2 | .993226 .1717066 -0.04 0.969 .7077736 1.393804

|

FAAR_K4 |

1978_1988 | .5588203 .1031999 -3.15 0.002 .389114 .8025415

1989_1999 | .4407643 .0918214 -3.93 0.000 .2930093 .6630272

2000_max | .3049773 .0739997 -4.89 0.000 .1895536 .4906853

|

fAge_KAT_K4 |

min-25 | .8045265 .1403361 -1.25 0.212 .5715609 1.132448

31-39 | 1.253828 .1827565 1.55 0.121 .9422533 1.66843

40-max | 1.015437 .2315634 0.07 0.946 .6494454 1.587683

|

MORS_ALDER_K5 |

min_19 | .8743413 .2085245 -0.56 0.573 .5478671 1.395362

25-29 | .9152681 .1442813 -0.56 0.574 .6719955 1.246609

30-34 | 1.275207 .2378491 1.30 0.192 .8847417 1.837999

35-max | 2.124915 .4535459 3.53 0.000 1.398491 3.22867

|

education_f |

primary | 1.261343 .1806621 1.62 0.105 .9526108 1.670132

secondary | .9595636 .1060966 -0.37 0.709 .7726068 1.191761

|

_cons | .0050767 .0013441 -19.95 0.000 .0030215 .0085298

------------------------+----------------------------------------------------------------

FAR_LOPENR |

var(_cons)| 1.680674 .3125811 1.167273 2.419885

-----------------------------------------------------------------------------------------

Note: Estimates are transformed only in the first equation to odds ratios.

Note: _cons estimates baseline odds (conditional on zero random effects).

LR test vs. logistic model: chibar2(01) = 33.49 Prob >= chibar2 = 0.0000

Regression S2.20. Paternal family history of premature stroke and neonatal death (subpopulation where paternal education is available and adjusted for education)

. melogit neo fam_stroke70_final_f_v2 i.FAAR_K4 i.fAge_KAT_K4 i.MORS_ALDER_K5 ib3.education_f if education_f!=. || FAR_LOPENR: , or

Fitting fixed-effects model:

Iteration 0: Log likelihood = -6180.2662

Iteration 1: Log likelihood = -1298.3416

Iteration 2: Log likelihood = -1262.7229

Iteration 3: Log likelihood = -1262.1332

Iteration 4: Log likelihood = -1262.1303

Iteration 5: Log likelihood = -1262.1303

Refining starting values:

Grid node 0: Log likelihood = -1287.2738

Fitting full model:

Iteration 0: Log likelihood = -1287.2738

Iteration 1: Log likelihood = -1269.7394

Iteration 2: Log likelihood = -1260.9075

Iteration 3: Log likelihood = -1260.6541

Iteration 4: Log likelihood = -1260.6536

Iteration 5: Log likelihood = -1260.6536

Mixed-effects logistic regression Number of obs = 67,175

Group variable: FAR_LOPENR Number of groups = 28,695

Obs per group:

min = 1

avg = 2.3

max = 10

Integration method: mvaghermite Integration pts. = 7

Wald chi2(13) = 35.35

Log likelihood = -1260.6536 Prob > chi2 = 0.0007

-----------------------------------------------------------------------------------------

neo | Odds ratio Std. err. z P>|z| [95% conf. interval]

------------------------+----------------------------------------------------------------

fam_stroke70_final_f_v2 | 1.01338 .2631148 0.05 0.959 .6092082 1.685695

|

FAAR_K4 |

1978_1988 | .7227309 .174429 -1.35 0.178 .4503411 1.159876

1989_1999 | .4262732 .1237431 -2.94 0.003 .2413193 .7529808

2000_max | .2609892 .1005434 -3.49 0.000 .1226608 .5553147

|

fAge_KAT_K4 |

min-25 | 1.089646 .2477508 0.38 0.706 .6978296 1.701459

31-39 | .9843849 .2249562 -0.07 0.945 .6289911 1.540584

40-max | 1.313561 .5211265 0.69 0.492 .6036076 2.85855

|

MORS_ALDER_K5 |

min_19 | .9206393 .2734238 -0.28 0.781 .514385 1.647748

25-29 | 1.010599 .2200732 0.05 0.961 .6595036 1.548606

30-34 | 1.083442 .3103714 0.28 0.780 .6179647 1.899536

35-max | .9952783 .3859148 -0.01 0.990 .4654734 2.128111

|

education_f |

primary | 1.679505 .3777827 2.31 0.021 1.080724 2.610045

secondary | 1.422121 .2608696 1.92 0.055 .9926464 2.03741

|

_cons | .0019232 .0008885 -13.54 0.000 .0007777 .0047563

------------------------+----------------------------------------------------------------

FAR_LOPENR |

var(_cons)| 1.269175 .6833078 .4418202 3.645841

-----------------------------------------------------------------------------------------

Note: Estimates are transformed only in the first equation to odds ratios.

Note: _cons estimates baseline odds (conditional on zero random effects).

LR test vs. logistic model: chibar2(01) = 2.95 Prob >= chibar2 = 0.0428

Regression S21. Paternal family history of premature stroke and perinatal death (subpopulation where paternal education is available adjusted for education)

. melogit totalloss_12aug fam_stroke70_final_f_v2 i.FAAR_K4 i.fAge_KAT_K4 ib3.education_f if education_f!=.|| FAR_LOPENR: , or

Fitting fixed-effects model:

Iteration 0: Log likelihood = -7531.4445

Iteration 1: Log likelihood = -3766.2955

Iteration 2: Log likelihood = -3686.2703

Iteration 3: Log likelihood = -3685.679

Iteration 4: Log likelihood = -3685.6786

Iteration 5: Log likelihood = -3685.6786

Refining starting values:

Grid node 0: Log likelihood = -3752.1778

Fitting full model:

Iteration 0: Log likelihood = -3752.1778

Iteration 1: Log likelihood = -3663.3516

Iteration 2: Log likelihood = -3661.9295

Iteration 3: Log likelihood = -3661.9229

Iteration 4: Log likelihood = -3661.9229

Mixed-effects logistic regression Number of obs = 67,648

Group variable: FAR_LOPENR Number of groups = 28,720

Obs per group:

min = 1

avg = 2.4

max = 10

Integration method: mvaghermite Integration pts. = 7

Wald chi2(9) = 41.93

Log likelihood = -3661.9229 Prob > chi2 = 0.0000

-----------------------------------------------------------------------------------------

totalloss_12aug | Odds ratio Std. err. z P>|z| [95% conf. interval]

------------------------+----------------------------------------------------------------

fam_stroke70_final_f_v2 | .9953869 .1465069 -0.03 0.975 .7459455 1.328241

|

FAAR_K4 |

1978_1988 | .6139279 .0900933 -3.32 0.001 .4604731 .8185221

1989_1999 | .4722699 .078853 -4.49 0.000 .340462 .6551066

2000_max | .3350453 .0671899 -5.45 0.000 .2261538 .4963673

|

fAge_KAT_K4 |

min-25 | .9006669 .1120555 -0.84 0.400 .7057695 1.149385

31-39 | 1.378258 .1500009 2.95 0.003 1.113502 1.705964

40-max | 1.506934 .2654186 2.33 0.020 1.067016 2.128226

|

education_f |

primary | 1.293663 .1569605 2.12 0.034 1.01987 1.640959

secondary | 1.021086 .0966384 0.22 0.825 .8482079 1.2292

|

_cons | .0070671 .0014114 -24.80 0.000 .004778 .0104528

------------------------+----------------------------------------------------------------

FAR_LOPENR |

var(_cons)| 1.559175 .2467673 1.143345 2.126242

-----------------------------------------------------------------------------------------

Note: Estimates are transformed only in the first equation to odds ratios.

Note: _cons estimates baseline odds (conditional on zero random effects).

LR test vs. logistic model: chibar2(01) = 47.51 Prob >= chibar2 = 0.0000

Regression S2.22. Paternal family history of premature stroke and stillbirth (subpopulation where paternal education is available and adjusted for marital status)

. melogit fetalloss_total fam_stroke70_final_f_v2 i.FAAR_K4 i.fAge_KAT_K4 i.MORS_ALDER_K5 i.mCivil if education_f!=. || FAR_LOPENR: , or

Fitting fixed-effects model:

Iteration 0: Log likelihood = -7018.4202

Iteration 1: Log likelihood = -2824.5042

Iteration 2: Log likelihood = -2794.3734

Iteration 3: Log likelihood = -2788.6699

Iteration 4: Log likelihood = -2788.6653

Iteration 5: Log likelihood = -2788.6653

Refining starting values:

Grid node 0: Log likelihood = -2839.0625

Fitting full model:

Iteration 0: Log likelihood = -2839.0625

Iteration 1: Log likelihood = -2772.006

Iteration 2: Log likelihood = -2771.7562

Iteration 3: Log likelihood = -2771.7561

Mixed-effects logistic regression Number of obs = 67,648

Group variable: FAR_LOPENR Number of groups = 28,720

Obs per group:

min = 1

avg = 2.4

max = 10

Integration method: mvaghermite Integration pts. = 7

Wald chi2(13) = 57.71

Log likelihood = -2771.7561 Prob > chi2 = 0.0000

-----------------------------------------------------------------------------------------

fetalloss_total | Odds ratio Std. err. z P>|z| [95% conf. interval]

------------------------+----------------------------------------------------------------

fam_stroke70_final_f_v2 | .9948831 .172038 -0.03 0.976 .7088918 1.396253

|

FAAR_K4 |

1978_1988 | .557603 .1033525 -3.15 0.002 .3877518 .8018562

1989_1999 | .4265129 .0885708 -4.10 0.000 .2839025 .6407594

2000_max | .2899914 .0699837 -5.13 0.000 .1807026 .465378

|

fAge_KAT_K4 |

min-25 | .8169085 .1431611 -1.15 0.249 .579432 1.151713

31-39 | 1.275513 .1859461 1.67 0.095 .958508 1.697361

40-max | 1.053334 .240166 0.23 0.820 .6737328 1.646815

|

MORS_ALDER_K5 |

min_19 | 1.012242 .248303 0.05 0.960 .6258709 1.637134

25-29 | .8737435 .1377243 -0.86 0.392 .6415239 1.190022

30-34 | 1.200337 .2227718 0.98 0.325 .8343111 1.726946

35-max | 2.005457 .4249452 3.28 0.001 1.323879 3.037933

|

mCivil |

unmarried | .6542795 .1333364 -2.08 0.037 .4388296 .9755076

wdo_divo_sep | 1.458428 .5824657 0.94 0.345 .6667034 3.190341

|

_cons | .0055126 .0014061 -20.39 0.000 .0033438 .0090879

------------------------+----------------------------------------------------------------

FAR_LOPENR |

var(_cons)| 1.690846 .3129507 1.176412 2.430239

-----------------------------------------------------------------------------------------

Note: Estimates are transformed only in the first equation to odds ratios.

Note: _cons estimates baseline odds (conditional on zero random effects).

LR test vs. logistic model: chibar2(01) = 33.82 Prob >= chibar2 = 0.0000

Regression S2.23. Paternal family history of premature stroke and neonatal death (subpopulation where paternal education is available and adjusted for marital status)

. melogit neo fam_stroke70_final_f_v2 i.FAAR_K4 i.fAge_KAT_K4 i.MORS_ALDER_K5 i.mCivil if education_f!=. || FAR_LOPENR: , or

Fitting fixed-effects model:

Iteration 0: Log likelihood = -6180.3307

Iteration 1: Log likelihood = -1298.9584

Iteration 2: Log likelihood = -1265.5942

Iteration 3: Log likelihood = -1265.0766

Iteration 4: Log likelihood = -1265.0735

Iteration 5: Log likelihood = -1265.0735

Refining starting values:

Grid node 0: Log likelihood = -1290.1951

Fitting full model:

Iteration 0: Log likelihood = -1290.1951

Iteration 1: Log likelihood = -1264.9219

Iteration 2: Log likelihood = -1264.0757

Iteration 3: Log likelihood = -1263.5244

Iteration 4: Log likelihood = -1263.5216

Iteration 5: Log likelihood = -1263.5216

Mixed-effects logistic regression Number of obs = 67,175

Group variable: FAR_LOPENR Number of groups = 28,695

Obs per group:

min = 1

avg = 2.3

max = 10

Integration method: mvaghermite Integration pts. = 7

Wald chi2(13) = 29.86

Log likelihood = -1263.5216 Prob > chi2 = 0.0049

-----------------------------------------------------------------------------------------

neo | Odds ratio Std. err. z P>|z| [95% conf. interval]

------------------------+----------------------------------------------------------------

fam_stroke70_final_f_v2 | 1.01355 .2631191 0.05 0.959 .609357 1.685848

|

FAAR_K4 |

1978_1988 | .7220971 .1745121 -1.35 0.178 .449658 1.159602

1989_1999 | .4279867 .123757 -2.93 0.003 .2428265 .7543352

2000_max | .2591379 .0994403 -3.52 0.000 .1221504 .5497522

|

fAge_KAT_K4 |

min-25 | 1.100715 .2516851 0.42 0.675 .7031412 1.723087

31-39 | .9903664 .2271281 -0.04 0.966 .6318056 1.552417

40-max | 1.350374 .5365572 0.76 0.450 .6197804 2.942187

|

MORS_ALDER_K5 |

min_19 | .950444 .2912517 -0.17 0.868 .5212984 1.732873

25-29 | .9615187 .20989 -0.18 0.857 .6268288 1.474914

30-34 | .9891952 .2830761 -0.04 0.970 .5645408 1.73328

35-max | .8919963 .3453439 -0.30 0.768 .4176507 1.905078

|

mCivil |

unmarried | 1.017877 .2457497 0.07 0.941 .6341418 1.633819

wdo_divo_sep | .5959542 .6020487 -0.51 0.608 .0822823 4.316376

|

_cons | .0026043 .0011449 -13.54 0.000 .0011002 .0061644

------------------------+----------------------------------------------------------------

FAR_LOPENR |

var(_cons)| 1.295022 .6779684 .464149 3.613243

-----------------------------------------------------------------------------------------

Note: Estimates are transformed only in the first equation to odds ratios.

Note: _cons estimates baseline odds (conditional on zero random effects).

LR test vs. logistic model: chibar2(01) = 3.10 Prob >= chibar2 = 0.0391

Regression S22.24. Paternal family history of premature stroke and perinatal death (subpopulation where paternal education is available adjusted for marital status)

. melogit totalloss_12aug fam_stroke70_final_f_v2 i.FAAR_K4 i.fAge_KAT_K4 i.mCivil if education_f!=.|| FAR_LOPENR: , or

Fitting fixed-effects model:

Iteration 0: Log likelihood = -7531.5458

Iteration 1: Log likelihood = -3766.8502

Iteration 2: Log likelihood = -3687.346

Iteration 3: Log likelihood = -3686.7233

Iteration 4: Log likelihood = -3686.7229

Iteration 5: Log likelihood = -3686.7229

Refining starting values:

Grid node 0: Log likelihood = -3753.1951

Fitting full model:

Iteration 0: Log likelihood = -3753.1951

Iteration 1: Log likelihood = -3664.119

Iteration 2: Log likelihood = -3662.7967

Iteration 3: Log likelihood = -3662.79

Iteration 4: Log likelihood = -3662.79

Mixed-effects logistic regression Number of obs = 67,648

Group variable: FAR_LOPENR Number of groups = 28,720

Obs per group:

min = 1

avg = 2.4

max = 10

Integration method: mvaghermite Integration pts. = 7

Wald chi2(9) = 39.90

Log likelihood = -3662.79 Prob > chi2 = 0.0000

-----------------------------------------------------------------------------------------

totalloss_12aug | Odds ratio Std. err. z P>|z| [95% conf. interval]

------------------------+----------------------------------------------------------------

fam_stroke70_final_f_v2 | .9962882 .1466357 -0.03 0.980 .7466265 1.329433

|

FAAR_K4 |

1978_1988 | .6002153 .0880034 -3.48 0.000 .4503022 .800037

1989_1999 | .449903 .0748266 -4.80 0.000 .3247502 .6232874

2000_max | .31335 .0624554 -5.82 0.000 .2120178 .4631131

|

fAge_KAT_K4 |

min-25 | .9455818 .1189851 -0.44 0.657 .7389075 1.210063

31-39 | 1.374514 .1490759 2.93 0.003 1.111298 1.700075

40-max | 1.524174 .2675612 2.40 0.016 1.080464 2.150101

|

mCivil |

unmarried | .7801556 .1182494 -1.64 0.101 .5796466 1.050024

wdo_divo_sep | 1.287241 .4792581 0.68 0.498 .6205049 2.670387

|

_cons | .0077893 .0014864 -25.44 0.000 .0053588 .0113221

------------------------+----------------------------------------------------------------

FAR_LOPENR |

var(_cons)| 1.564496 .2466024 1.148695 2.130809

-----------------------------------------------------------------------------------------

Note: Estimates are transformed only in the first equation to odds ratios.

Note: _cons estimates baseline odds (conditional on zero random effects).

LR test vs. logistic model: chibar2(01) = 47.87 Prob >= chibar2 = 0.0000

.

end of do-file

.
