# Supplementary material for: Paternal family history of premature atherosclerotic disease and perinatal death: A population-based cohort study
Source: PLoS One. 2025 Jan 8;20(1):e0313821. doi: 10.1371/journal.pone.0313821 (PMC11709281; doi:10.1371/journal.pone.0313821)
Supplement: Attachment — Submitted filename: Response to Reviewers.docx [file pone.0313821.s003.docx]

We reply to each comment in point-by-point fashion. Our responses are shown as “Response”. Page and line numbers in the rebuttal letter correspond to the document named “Revised Manuscript with Track Changes” while all tracked changes are shown.

1. An acknowledgment of the limitations of the study in the abstract.

Response: Thank you for pointing this out. Major limitations of the study were added to the abstract. See Page 3, Line 51-52:

“Limitations: Socioeconomic background of fathers was captured using imperfect proxy. Potential misclassification of family history and selection bias should be considered.”

2. Eligibility criteria for the different cohort participants that were used for this study need to be added.

Response: See Page 12, Line 269-272

“Eligibility criteria are fathers who had participated in at least one health survey before the age of 50. If a father participated in more than one survey, we used the survey nearest to the birth of the child (referred to here as ‘the relevant health survey’)”

3. Describe efforts to address potential sources of bias.

Response: Possible sources of bias in our study as mentioned in limitations (Page 39-42, Line 694-759) are selection bias infringed in the traditional survey research, information bias due to misclassification of having family history or not and residual confounding.

See page 39-40, lines 692-703 where we have discussed potential selection bias:

“Since fathers chose whether to participate in the health surveys, a potential self-selection bias must be considered. Having a family history of CVD might be a motivation for fathers to participate in a health survey where one focus was CVD. However, since CVD in a father’s family was (and is) not an acknowledged risk factor for perinatal loss, participation is likely not related to both a family history of CVD and the experience of a perinatal loss. The prevalence of perinatal loss in the total study population was also similar to that in the total Norwegian population during the study years.^52^ Moreover, even in a situation with self-selection into the study population, this does not necessarily affect the relation between a given exposure and a given outcome. A study evaluating selection bias in the Mother, Father and Child cohort study in Norway, have concluded that in spite of a participation rate around 40%, higher socioeconomic level in participants than non-participants with bias in prevalence estimates of exposures and outcomes, the studied exposure-outcome associations were valid.^53^”

See page 41, lines 717-724 where we discussed potential misclassification bias:

“Further, misclassification of exposure is another potential limitation. The relationship between paternal family history of atherosclerotic diseases and perinatal death was unknown at the time of survey and thus this misclassification is likely non-differential^54^ which could attenuate our results. A large non-differential misclassification could then be a factor explaining our null findings. Errors in reporting of family history can occur when individuals are unaware of their relatives' disease status or when their relatives themselves are unaware of their own health status, thus leading to misclassification of the exposure.^55^ However, a study that investigates concordance of family history of CVD between offspring and parents found that family history provided by young and middle-aged Norwegian stroke patients is in good concordance with parental report of their own CVD status.^56^ A high proportion of missing in family history could also lead to misclassification. However, we have a small amount of missing data (less than 1%) in both family history of CHD and stroke. To sum up, we can conclude that the potential non-differential misclassification of family history in our study is likely small and not enough to explain our null findings.”

See page 42, lines 742-753 where we discussed residual confounding due to missing data on socioeconomic level:

“For studying the effect of paternal family history on perinatal death, it is relevant to adjust for the fathers' socioeconomic background. Unfortunately, we lacked that information and had large proportions of missing data for father’s own education, a proxy for his socioeconomic background. We did, however, have information on marital status (being married or having a partner) at the time of childbirth and age at childbirth which may capture some social factors, and we therefore included these as proxy variables for father’s socioeconomic background. We also conducted separate analyses for the subpopulation where information on father’s education was available and got almost the same estimates and 95% CI when adjusting for father’s age and education as when adjusting for father’s age and marital status. Therefore, we argue that using age and marital status is a satisfactory way to deal with missing information on father’s education. Nonetheless, we still cannot exclude residual confounding by other unknown factors.”

4. How were missing data addressed?

Response: To address editor’s concern on missing data, we added a section on missing data in the methods. Page 18, Line 395-403:

“There were no missing data on outcome and adjustment variables. There were 12 missing values for family history of CHD (0.0 % on 512,113 of total observations) and 1,238 for family history of stroke (0.61% on 204,495 of total observations). The reason for having different number of observations between family history of CHD and stroke is that some surveys did not collect family history of stroke at all, and we thus had to use two separate study populations. Analyses were done separately for family history of CHD and family history of stroke, and we did complete case analyses in these two populations.”

5. Discuss limitations of the study in more detail, taking into account sources of potential bias or imprecision. Discuss both direction and magnitude of any potential bias.

Response: In the revised version, we expanded the ‘Limitations’ (Page 39-42, Line 687-753) to accommodate the Editor’s concerns with regards to sources of bias and imprecision. See page 39-40, lines 692-703 where we have discussed potential selection bias, see page 41, lines 717-734 where we discussed potential misclassification bias, see page 42, lines 742-753 where we discussed residual confounding due to missing data on socioeconomic level, see page 41, line 735-740 where we discussed imprecision.

Reviewer #1: Review on the manuscript “Paternal family history of premature atherosclerotic disease and perinatal death: a population-based cohort study”

Thank you very much for providing me the opportunity to review this manuscript. The study provides a detailed overview of a population-based cohort study investigating the association between paternal family history of premature atherosclerotic disease and perinatal death. The study fills a gap in the literature regarding non-modifiable paternal risk factors and perinatal death, contributing valuable insights to the field.

Abstract: Well-written

Introduction:

The introduction effectively conveys the information by providing a comprehensive background on perinatal mortality, defining key terms, and discussing the multifactorial causes of perinatal death. It also highlights the significance of paternal factors, particularly genetic influences, which have been less studied compared to maternal factors. The identification of a research gap concerning the association between paternal family history of premature atherosclerotic diseases and perinatal outcomes is clear and compelling. However, the introduction could be improved by breaking down the information into more concise sections or paragraphs to enhance readability. There is a need to focus more on the study's rationale and objectives to make it more engaging. Incorporating a brief summary of the key points at the end could help reinforce the study's purpose and significance.

Response: Based on the reviewer comment, we broke down the introduction into concise paragraphs and highlighted the study’s rationale and objectives. A summary of key point was also added at the end (Page 9 Line 193-205).

“To sum up, previous studies have documented associations between several sociodemographic, lifestyle and environmental factors in fathers and the risk of perinatal loss. Further, paternal factors have been found to influence the risk of pregnancy complications associated with perinatal loss. The risk of pregnancy loss increased with increasing number of cardiovascular risk factors in fathers, this could be explained by common underlying aetiologic factors. Family history of cardiovascular disease reflects genetic susceptibilities, shared environment, and common behaviors and may be used to highlight potential common underlying aetiologic mechanisms for both cardiovascular risk and perinatal loss. So far, no studies have focused paternal family history of cardiovascular disease and the risk of perinatal loss. This study, therefore, aimed at exploring the potential association between paternal family history of premature coronary heart disease or stroke and stillbirth, neonatal death, and perinatal death that may share common underlying aetiologic mechanisms.”

Materials and Methods Review

The "Materials and Methods" section of the study is comprehensive and well-structured, providing clear descriptions of data sources, exposures, outcomes, and statistical analyses. Nevertheless, there are some weaknesses which can improve

the readability and quality of the manuscript.

1. At some places provided information seems to be dense and might be overwhelming for some readers. Breaking it into more concise paragraphs and adding subheadings could improve readability. For example, introduce subheadings for sections like "Data Sources," "Linkage and Inclusion Criteria," "Definitions," "Statistical Analysis," and "Ethical Considerations." This will make the section more navigable.

Response: Thank you for pointing this out. Subheadings are added as suggested in methods.

2. The data spans from 1974 to 2003; changes in medical practices and lifestyle factors over these decades might influence the outcomes and their interpretation in the current context.

Response: We totally agree with reviewer that medical practices and lifestyle factors have changed over these decades, possibly leading to better care and reduction of perinatal mortality. We have therefore adjusted all analyses for year of birth (also the “crude” analyses – Model 1) to avoid the potential temporal effect of these changes in our associations (Page 17, line 374-375). Further, we tested whether there is an interaction between exposure and year of childbirth and did not find one (P for interaction = 0.531)

3. The lack of socioeconomic data and high missing rates for father's education are noted as limitations. Addressing how these missing data might impact the study's findings could provide a more balanced view.

Response: To address the reviewer’s concern, we conducted separate analyses for the subpopulation where information on father’s education was available (13.2% of the study population) and updated in the method section. Page 17, Line 390-393.

The results of the analyses (adjusting for age at birth and paternal education) in this subgroup, gave almost the same estimates and 95% CI as when the analyses were adjusted for age at birth and marital status (as done in the main analyses). Page 29, Line 542-547 and tables at Supporting Information1, Table S5 and S6.

We have also added a comment on this in the Discussion part, Limitations, page 42, lines 742-753.

“For studying the effect of paternal family history on perinatal death, it is relevant to adjust for the fathers' socioeconomic background. Unfortunately, we lacked that information and had large proportions of missing data for father’s own education, a proxy for his socioeconomic background. We did, however, have information on marital status (being married or having a partner) at the time of childbirth and age at childbirth which may capture some social factors, and we therefore included these as proxy variables for father’s socioeconomic background. We also conducted separate analyses for the subpopulation where information on father’s education was available and got almost the same estimates and 95% CI when adjusting for father’s age and education as when adjusting for father’s age and marital status. Therefore, we argue that using age and marital status is a satisfactory way to deal with missing information on father’s education.”

Table S5: Association between paternal family history of premature coronary heart disease and subsequent risk of perinatal losses (restricted to the subpopulation with available data in father’s education)

| Family history of premature CHD | | Total | Deaths | ^Σ^Odds Ratio (95% CI) | |
| --- | --- | --- | --- | --- | --- |
|  |  |  | N (%) | Model 1* | Model 2** |
| Stillbirth | | | | | |
|  | No | 58,070 | 413 (0.7) | 1.00 (Ref) | 1.00 (Ref) |
|  | Yes | 9,578 | 60 (0.6) | 0.82 (0.62; 1.09) | 0.83 (0.62; 1.11) |
| Neonatal Death | | | | | |
|  | No | 57,657 | 158 (0.3) | 1.00 (Ref) | 1.00 (Ref) |
|  | Yes | 9,518 | 28 (0.3) | 0.96 (0.64; 1.45) | 0.97 (0.65; 1.47) |
| Perinatal Death | | | | | |
|  | No | 58,070 | 571 (1.0) | 1.00 (Ref) | 1.00 (Ref) |
|  | Yes | 9,578 | 88 (0.9) | 0.87 (0.68; 1.10) | 0.88 (0.69; 1.12) |

^Σ^Multilevel mixed effect logistic regression with random intercepts by father’s identification number. The birth is an observation unit

*Adjusted for birth year, paternal and maternal age at childbirth, and *paternal education*

**Adjusted for birth year, paternal and maternal age at childbirth, and *marital status*

Table S6: Association between paternal family history of stroke and subsequent risk of perinatal losses (restricted to the subpopulation with available data on father’s education)

| Family history of stroke | | Total | Deaths | ^Σ^Odds Ratio (95% CI) | |
| --- | --- | --- | --- | --- | --- |
|  |  |  | N (%) | Model 1* | Model 2** |
| Stillbirth | | | | | |
|  | No | 61,888 | 432 (0.7) | 1.00 (Reference) | 1.00 (Reference) |
|  | Yes | 5,760 | 41 (0.7) | 0.99 (0.71; 1.39) | 0.99 (0.71; 1.40) |
| Neonatal Death | | | | | |
|  | No | 61,456 | 169 (0.3) | 1.00 (Reference) | 1.00 (Reference) |
|  | Yes | 5,719 | 17 (0.3) | 1.01 (0.61; 1.69) | 1.01 (0.61; 1.69) |
| Perinatal Death | | | | | |
|  | No | 61,888 | 601 (1.0) | 1.00 (Reference) | 1.00 (Reference) |
|  | Yes | 5,760 | 58 (1.0) | 1.00 (0.75; 1.33) | 1.00 (0.75; 1.33) |

^Σ^Multilevel mixed effect logistic regression with random intercepts by father’s identification number. The birth is an observation unit.

Adjusted for birth year, paternal and maternal age at childbirth, and *paternal education*

**Adjusted for birth year, paternal and maternal age at childbirth, and *marital status*

4. Incorporate visual aids, such as flowcharts or diagrams (like the directed

acyclic graph mentioned), to illustrate data linkages and the analysis process. This can help in understanding complex methodologies.

Response: Thank you for pointing this out. Illustration of data linkages is added as Figure 1. See page 12, line 277 and 278

**Family History of premature CHD**

(N=512,113)

- Screening of 40-year-old program (1985-1999)

n = 380,206 (74.2%)

- Three County Health Survey (1974-1988)

n = 54,686 (10.7%)

- Cohort of Norway (1994-2003)

n = 77,219 (15.1%)

- Missing =12 (0.0%)

**Perinatal death**

Medical Birth Registry of Norway

(1967-2020)

Total births N = 512,113

Stillbirth = 4,321 (0.8%)

Neonatal Death = 2,383 (0.5%)

Perinatal Death = 6,704 (1.3%)

**Family History of premature Stroke**

(N= 204,495) *

- Screening of 40-year-old program (1985-1999)

n = 126,038 (61.6%)

- Three County Health Survey (1974-1988)

n = 0

- Cohort of Norway (1994-2003)

n = 77,219 (37.8%)

- Missing = 1,238 (0.6%)

**Perinatal death**

Medical Birth Registry of Norway

(1967-2020)

Total births N= 204,495

Stillbirth = 1,371 (0.7%)

Neonatal Death = 682 (0.3%)

Perinatal Death = 2,053 (1.0%)

*Some health surveys do not collect family history of stroke at all

Figure1. Data sources, linkages and number of observations

Results

The "Results" section presents a clear and comprehensive overview of the findings from the study. However, there are some areas for improvement to enhance clarity and readability.

1. The section lacks detailed explanations and interpretations of the observed differences in maternal characteristics. Provide more interpretation and discussion on the observed differences in maternal characteristics and their potential implications for the study's findings.

Response: According to our DAG (Figure 2), paternal factors associate with maternal factors that could be the mediators in examining the association between the exposure and outcome. We mentioned that maternal factors are relevant to our study in the method section (page 14, line 321-325):

“When evaluating the association between family history of atherosclerotic disease and perinatal death, focusing on the total effect of paternal family history, paternal and maternal own risks factors such as hypertension, diabetes, total cholesterol, smoking, physical activity as well as infant factors such as birth weight, gestational age, SGA 2.5 percentile, foetal anomalies will be mediators (Figure 2), and should not be adjusted for.”

To address the reviewer’s concern on impact of maternal characteristics on finding of our study, we have now done additional analyses after excluding mothers with known risk factors for perinatal death. See Method section, page 15, line 326-330:

“Nonetheless, to differentiate the effect of paternal family history from maternal known risk factors for perinatal death, we did additional analyses where mothers with some important risk factors for perinatal death such as preeclampsia including HELLP syndrome and eclampsia, pregestational and gestational hypertension, and pregestational and gestational diabetes were excluded.”

See result section, page 29, line 540-541: “The resulting point estimates and 95% CI were almost the same as in the total population”. The resulting point estimates and 95% CI were presented in Supporting tables: Supporting Information 1, Table S3 and S4.

Table S3. Association between paternal family history of premature coronary heart disease and subsequent risk of perinatal death (excluding mothers with known risk factors of perinatal death) ^⊥^

| Family history of premature CHD | | Total | Deaths | ^Σ^Odds Ratio (95% CI) * |
| --- | --- | --- | --- | --- |
|  |  |  | N (%) |  |
| Stillbirth | | | | |
|  | No | 427,279 | 3,484 (0.8) | 1.00 (Reference) |
|  | Yes | 59,507 | 466 (0.8) | 1.01 (0.91; 1,12) |
| Neonatal Death | | | | |
|  | No | 423,795 | 1,980 (0.5) | 1.00 (Reference) |
|  | Yes | 59,041 | 256 (0.4) | 0.99 (0.86; 1.13) |
| Perinatal Death | | | | |
|  | No | 427,279 | 5,464 (1.3) | 1.00 (Reference) |
|  | Yes | 59,507 | 722 (1.2) | 1.00 (0.92; 1.09) |

^⊥^Excluding mothers with preeclampsia including HELLP syndrome and eclampsia, pregestational and gestational hypertension, pregestational and gestational diabetes

*Adjusted for year of birth, paternal [≤ 25, 26 – 30 (reference), 31 – 39, ≥ 40 yr] and maternal age [ ≤ 19, 20-24 (reference), 25 – 29, 30 – 34, ≥ 35] at childbirth, marital status married/have partner (reference), unmarried, widowed/divorced/ separated]

^Σ^Multilevel mixed effect logistic regression with random intercepts by father’s identification number. The birth is an observation unit.

Table S4. Association between paternal family history of premature stroke and subsequent risk of perinatal death (excluding mothers with known risk factors of perinatal death) ^⊥^

| Family history of premature CHD | | Total | Deaths | ^Σ^Odds Ratio (95% CI) * |
| --- | --- | --- | --- | --- |
|  |  |  | N (%) |  |
| Stillbirth | | | | |
|  | No | 174,534 | 1,173 (0.7) | 1.00 (Reference) |
|  | Yes | 17,696 | 116 (0.7) | 0.98 (0.80;1.20) |
| Neonatal Death | | | | |
|  | No | 173,361 | 575 (0.3) | 1.00 (Reference) |
|  | Yes | 17,580 | 66 (0.4) | 1.09 (0.84; 1.43) |
| Perinatal Death | | | | |
|  | No | 174,534 | 1,748(1.0) | 1.00 (Reference) |
|  | Yes | 17,696 | 182 (1.03) | 1.01 (0.86;1.19) |

^⊥^Excluding mothers with preeclampsia including HELLP syndrome and eclampsia, pregestational and gestational hypertension, pregestational and gestational diabetes

*Adjusted for year of birth, paternal [≤ 25, 26 – 30 (reference), 31 – 39, ≥ 40 yr] and maternal age [ ≤ 19, 20-24 (reference), 25 – 29, 30 – 34, ≥ 35] at childbirth, marital status married/have partner (reference), unmarried, widowed/divorced/ separated]

^Σ^Multilevel mixed effect logistic regression with random intercepts by father’s identification number. The birth is an observation unit.

We have further elaborated on the description of maternal characteristics in the result section (page 26, line 470-490):

“Family history of CHD

The mothers whose partners had family history of CHD were slightly younger when delivering (26.7 vs 26.9 years) and had a slightly higher prevalence of preeclampsia (2.9% vs 2.6%) compared to those whose partners did not have family history of CHD, but differences were very small. Only a small proportion of them had chronic hypertension (0.2%), pregestational (0.2%) and gestational diabetes (0.2%), preeclampsia including HELLP syndrome and eclampsia (2.7%). Again, the differences between those with and without family history were small. Parity and the proportions of placenta previa and abruptio placenta were the same between the two group of mothers.

Family history of Stroke

Mothers whose partners had family history of stroke were slightly younger at the time of childbirth compared to their counterparts (27.3 vs 27.5 years). Parity, the proportions of mothers with chronic hypertension (0.3%), pre-gestational (0.3%) and gestational diabetes (0.4%), and pre-eclampsia (2.9), placental previa (0.2) and abruption (0.6) were almost the same in the groups with and without family history.”

The implications of maternal characteristics on the study findings were added to the discussion (page 38, 655-658).

“Regarding maternal characteristics the differences between those whose partners had or did not have family history (for instances age, hypertension and diabetes) were small However, maternal age was adjusted in model 2 and we did additional analyses excluding mothers with some important risk factors for perinatal loss, and results were basically the same.”

2. The section provides limited context on why the maternal characteristics are important in relation to the study's main focus on paternal family history and perinatal death.

Response: Please see our response to reviewer’s question on Results section, question number 2 (see above).

3. The section lacks interpretation of the findings, such as why there may be no significant association between paternal family history of CHD and perinatal death.

Response: Interpretation of the findings is added to the Discussion section, page 36-38, line 620-658. Please also see our response to reviewer’s question 1 on Discussion section (see below).

4. The reporting of percentages and means could be clearer and more consistent to improve readability. Ensure consistent and clear reporting of percentages and means and consider using additional visual aids to improve clarity.

Response: Percentages and means are updated to be consistent (Page 20, line 445, page 21, line 447,448, line 454, 455, 458).

Discussion

The discussion section effectively summarizes the key findings of the study, situating them within the broader context of existing research. However, there are several areas where the discussion could be strengthened to enhance clarity, depth, and engagement.

1. The discussion lacks in-depth analysis and interpretation of why the expected associations were not observed and what this implies for future research. Provide a more detailed analysis of the potential reasons for the lack of significant associations. Discuss the possible impact of measurement timing, residual confounding, and misclassification of family history.

Response: Potential reasons for the lack of significant associations are added in addition to genetic factors to the Discussion (Page 36-38, Line 620-658)

We discussed on genetic factors acting through mother and father (page 36, line 623-629), subset of pregnancy loss (page 36, line 630-633),non-differential misclassification and power of the study (page 36-37, line 633-636), minimal difference of risk factors (the mediators between the paternal family history and perinatal death) between those with family history (page 37, line 637-654) and those without, and the impact of maternal characteristics (page 37, line 655-658).

2. The discussion could benefit from a deeper engagement with the broader implications of the findings, especially concerning genetic and environmental factors. Elaborate on the broader implications of the findings for understanding genetic and environmental contributions to perinatal outcomes. Discuss how these results can inform future research directions.

Response: Implications of the findings and future result direction is added as reviewer suggested. See Page 42-43 Line 755-779:

“Family history gives information about genetic and persisting environmental risk factors for many diseases. Taking a family history has low cost and is an acceptable and informative first step to identify individuals from the general population who may benefit from genetic testing. Some studies found association between maternal family history of CVD and pregnancy loss or stillbirth. Although we did not find association between paternal history of premature atherosclerotic disease and perinatal death, we did find that fathers with a family history of premature atherosclerotic disease were more likely to smoke, be obese and have higher blood pressure and higher cholesterol than fathers without a family history. A study reported that the risk of pregnancy loss increased with increasing number of cardiovascular risk factors in fathers. Our study was not able to relate these factors to the risk of perinatal loss since most of the fathers measured these factors in health surveys after the birth, often with a long-time lag. It does, however, suggest that family history of premature atherosclerotic disease might be an indicator of the fathers’ underlying health that could be optimised during preconception period. It also calls for more studies where the relation between these cardiovascular risk factors in fathers can be studied in relation to future risk of perinatal loss.

We also found that infants born to fathers with family history of CHD had lower mean birth weight, were more likely to be born preterm, extremely preterm, being SGA and more likely to have congenital anomalies than those without family history. We therefore warrant larger population-based studies to replicate and confirm the findings of the current study, also in low- and middle-income countries where both atherosclerotic risk factors and perinatal deaths are higher than in the Norwegian population. Further studies on how the experience of a perinatal death may affect fathers’ health and risk factors later in life would be of interest as well. The perinatal care clinicians could collect information on family history of premature atherosclerotic disease in both parents to identify those who might benefit from closer follow up during pregnancy.”

3. The flow could be improved by better organizing the comparison with other studies and making clearer transitions between different sections of the discussion. Reorganize the discussion to improve coherence. Start with a summary of the key findings, followed by a comparison with existing literature, then move on to strengths and limitations, and end with broader implications and future directions.

Response: The flow is re-organized starting with summary of key findings as the first paragraph (page 34, lines 574-578), comparison with existing literature (page 34-35, lines 580-600), in-depth analysis and interpretation of why the expected associations were not observed [genetic factors acting through mother and father (page 36, line 623-629), subset of pregnancy loss (page 36, line 630-633),non-differential misclassification and power of the study (page 36-37, line 633-636), minimal difference of risk factors (the mediators between the paternal family history and perinatal death) between those with family history (page 37, line 637-654) and those without, and the impact of maternal characteristics (page 38, line 655-658)], strengths and limitations (Page 39-42, Line 681-753), and then ends with broader implications and future directions (page 42-43, lines 754-779).

Conclusion

The conclusions section is well-written. However, there are areas where the conclusion can be enhanced to provide a more comprehensive and impactful summary.

1. The conclusion could benefit from more depth and specificity regarding the implications of the findings and the observed risk factors. Provide more detail on the implications of the findings for clinical practice and public health, especially

regarding paternal health screening and intervention.

Response: Our implication is that family history of premature atherosclerotic diseases could be a quick screening tool for underlying health that may affect pregnancy outcomes (page 44, line 790-792)

2. Place the findings in a broader context by discussing their relevance to other studies and the current understanding of perinatal health risks.

Please see our response to reviewer’s question 2 in discussion section.

3. If applicable, suggest any policy recommendations or changes in clinical practice that could arise from the findings.

Response: We suggested a policy recommendation that preconception and perinatal care clinicians might want to ask family history of premature atherosclerotic diseases in both parents as a cost-effective screening tool for underlying health of both parents. (page 44, line 790-792)

Reviewer #2: 3 comments

-Until 1975, for the WHO, the limit of viability was 1000 g and 28 weeks. After this date, the theoretical definition of a birth living was the following: “the complete expulsion or extraction of the body of the mother of a product of conception weighing at least 500 grams (or a gestational age of 22 years weeks). Lines 99-101?

Response: The definition was update according to the reviewer’s comment. See page 5, line 110-114.

“Until 1975, the World Health Organization (WHO) defined the stillbirth rate as the number of babies born with no signs of life weighing 1000 grams or more or being born at 28 gestational weeks or more per 1000 total births.^1^ After that, stillbirth is defined as the expulsion of a foetus with no signs of life weighing 500 grams or more at 22 weeks or more of gestation.”

Model 2 adjusting for birth year of child, paternal and maternal age at childbirth and paternal marital status. Why paternal marital status? Marital status is an unsuitable proxy for socio-economic factor

Response. Please see our answers to Reviewer 1, Materials and Methods, point 3, where we discuss the problem of lacking data on socioeconomic factors.

Ideally, we would have wanted data on the fathers’ socioeconomic background (see our DAG, Figure 2), which we did not have. Father’s own education could be a proxy for his SES background, but we only had this information for a small proportion of fathers. We therefore chose to use age at childbirth and marital status as proxies and we tested this in the subset of fathers where we had data on father’s education (Supplemental Information 1, Table S5 and S6).

We agree that marital status is not a perfect proxy for social status.

However, a review by Benjain R. Karney^1^ reported that, couples of higher social levels postpone marriage and childbirth to invest in education and careers, but eventually marry at a high rate and have relatively low risk of divorce. Couples with low socioeconomic level are more likely to cohabit and give birth prior to marriage and less likely to marry at all. We found that fathers with family history of premature atherosclerotic diseases were more likely to be unmarried (9.9% vs 9.2%) and more likely to be widowed/divorced/separated (9.6% vs 9.2%). Moreover, they were more likely to have children at younger age (18-25 year). Among the fathers where we had data on education, fathers with a family history were less likely to have tertiary education compared to fathers without a family history (33.1% vs 35.5%). Therefore, we decided to use age and marital status as a proxy of socio-economic level.

Even after reading your study, I'm doubtful in terms of pathophysiology of the link that you tried to highlight between paternal family history of atherosclerotic diseases and offspring perinatal death. But the study is easy to read and well structured

Response: As the reviewer pointed out, there is no known pathophysiology that links between paternal family history of atherosclerotic disease and offspring perinatal death to the best of our knowledge.

However, pregnancy losses (miscarriage and stillbirth) are associated with ischaemic disease in both heart and brain, and probably also with the wider range of atherosclerotic diseases. These associations are seen not only at the level of the individual woman but also within families. See Discussion, page 35, line 596-600. This suggests that some types of pregnancy loss may share an underlying aetiologic mechanism (which potentially has a genetic or epigenetic component) with atherosclerotic disease, and that in families with ischaemic and/or atherosclerotic disease, women (or man) may be predisposed to miscarriage and stillbirth.

Based on previous studies’ findings on paternal effects on placental vascular dysfunction related to pregnancy complications and familial aggregation of these findings (see Introduction, page 7, line 162-170), we hypothesized that children (fathers in our study) to parents with CVD may be predisposed to pregnancy losses. Family history of premature coronary heart disease (CHD)_ a non-modifiable risk factor_ reflects genetic susceptibilities, shared environment, and common behaviours. Genetic susceptibilities could include factors such as haemostatic dysfunction and genes responsible for vascular dysfunction that are assumed to have shared underlying aetiologic mechanism between pregnancy loss or complications during pregnancy and CHD.

Reference

1.	Karney BR. Socioeconomic Status and Intimate Relationships. Annu Rev Psychol. 2021 Jan 4;72:391–414.
